# Many-Objective Optimisation of Global Quantum Internet Topologies: A Compact Parametric Representation with an Analytical BDCZ Oracle

**DOI:** 10.3390/e28091041

**Published:** 2026-09-21

**Authors:** Jesús Gil Ruiz, Diego Rubén Rodríguez Regadera, Rafael Muñoz Gil

**Affiliations:** 1 Science and Aerospace Department, Universidad Europea de Madrid, Calle Tajo s/n, Villaviciosa de Odón, 28670 Madrid, Spain; diegoruben.rodriguez@universidadeuropea.es (D.R.R.R.); rafael.munoz@universidadeuropea.es (R.M.G.); 2 Universidad Alfonso X el Sabio, Villanueva de la Cañada, 28691 Madrid, Spain; 3 Universidad Internacional de Valencia (VIU), 46002 Valencia, Spain

**Keywords:** quantum internet, many-objective optimisation, evolutionary multi-objective optimisation, topology design, BDCZ repeater protocol, hypervolume

## Abstract

We address the design of global quantum internet topologies that jointly optimise four physical objectives: average end-to-end Werner-state fidelity, entanglement rate, latency and coverage, evaluated with an analytical BDCZ oracle whose path selection is exact under the stated Werner-composition model and whose satellite links respect a single-satellite visibility limit. The search space is heterogeneous (fibre, satellite, free-space optical), comprising 1002=4950 possible edges over 100 cities; direct per-edge categorical optimisation degenerates. We propose a mixed representation of ten physical design parameters plus one stochastic realisation index (eleven decision variables in total) decoding into a topology via *k*-nearest-neighbour rules. Seven many-objective algorithms (NSGA-II, NSGA-III, MOEA/D, SMS-EMOA, RVEA, SPEA2, AGE-MOEA-II) are compared at a matched budget over 30 seeds, scored against a shared reference front. NSGA-II attains the highest mean hypervolume (0.353±0.040) but is statistically indistinguishable from SPEA2 and AGE-MOEA-II (0.352, 0.347); after Holm correction across all 63 tests, this top group separates significantly from NSGA-III, SMS-EMOA, RVEA and MOEA/D, though NSGA-III and SMS-EMOA remain indistinguishable in HV/IGD (spacing unresolved). NSGA-II’s lead is driven by 6 of its 30 seeds reaching a higher-HV region, a bimodality we report. An eight-variant decoder ablation (NSGA-III) shows fibre is necessary while satellite’s contribution is not detectable, and one classical constructor (a Random Geometric Graph) remains non-dominated; none of the 561 front points connects all 100 cities under the satellite model. On four additional real-city instances (five algorithms), MOEA/D is last throughout; SMS-EMOA/RVEA ranks vary by instance, and NSGA-II/NSGA-III reverses, without significance, twice; the fibre transmittance law is cross-checked against SeQUeNCe (Spearman ρ=1.0). The pooled front is released as QI-Bench-Pareto-v1.

## 1. Introduction

Quantum communication networks promise unconditionally secure key distribution, distributed quantum computing and high-precision clock synchronisation [1,2,3,4]. While metropolitan-scale prototypes have been operational for several years (the Beijing–Shanghai backbone [5], the Cambridge testbed [6], the Tokyo QKD network [7], the Vienna QKD network [8]), *global-scale* quantum networks remain an open design problem. The fundamental difficulty is the combination of four constraints. First, end-to-end fidelity decays exponentially with the number of repeater hops [9,10,11], favouring sparse topologies. Second, entanglement-distribution rates scale inversely with hop count via the bottleneck of slowest links and swap-success probabilities [12,13]. Third, latency is the sum of per-link propagation times, sensitive to geographical layout. Fourth, coverage—the fraction of city pairs reachable above a fidelity threshold—demands dense connectivity. These four objectives interact in non-trivial ways and are formally optimised jointly throughout this paper; Section 7 shows the discovered front’s *effective* dimensionality is nonetheless substantially lower than four, which we return to when discussing why dominance-and-density algorithms designed for two or three objectives (NSGA-II, SPEA2) perform at least as well here as methods built for higher-dimensional many-objective fronts.

Prior topology design work for quantum networks has predominantly addressed single-objective formulations: entanglement-distribution rate optimisation along a repeater chain [14], genetic optimisation applied to the inverse problem of inferring a network’s topology from indirect measurements [15], or reinforcement-learning routing within fixed topologies [16]. Multi-objective formulations exist for classical networks [17] but have not been instantiated for the quantum case at intercontinental scale.

A naive instantiation of NSGA-III over a per-edge categorical representation (one {no edge, fibre, satellite, FSO} variable per candidate edge, 4950 variables in our setting, Section 6.3) produces degenerate Pareto fronts of fewer than 20 points after 80 generations: a known pathology of evolutionary search in high-dimensional categorical spaces with structured fitness landscapes. We resolve this by introducing an *eleven-dimensional mixed (seven continuous, four integer) parametric representation* that decodes deterministically into a heterogeneous topology via *k*-nearest-neighbour rules with per-medium inclusion probabilities. The representation is interpretable (each dimension corresponds to a physical design parameter) and reduces the search dimension by two orders of magnitude.

### Contributions

1. A compact 11-parameter mixed (continuous/integer) representation for global quantum network topologies, with a deterministic decoder producing valid heterogeneous fibre/satellite/FSO graphs over 100 cities (Section 4).
2. Reuse, as a direct evolutionary fitness function rather than as a source of training labels, of the vectorised analytical BDCZ oracle first introduced in the companion QI-Bench-100 benchmark [18], which computes all four end-to-end objectives on a single CPU (Section 4.2).
3. A systematic comparison of seven algorithms—NSGA-II, NSGA-III, MOEA/D, SMS-EMOA, RVEA, SPEA2 and the 2022 AGE-MOEA-II—with 30 random seeds each, all scored against a single shared reference front and normalisation, reporting mean ± std for hypervolume, IGD, spacing, cardinality and runtime (plus HV’s median, given the bimodality below; the full mean/median/IQR breakdown for all four metrics is released with the reproducibility package), together with a Kruskal–Wallis omnibus test and Holm-corrected pairwise Mann–Whitney tests over the whole 63-test family (independent samples, the primary analysis), cross-checked against a common-seed paired (Friedman/Wilcoxon) sensitivity analysis and a random-search sanity check (Section 6.1).
4. An eight-variant, 30-seed ablation of the decoder isolating the contribution of each link type and structural prior, including satellite-only, satellite+FSO and FSO-only variants (Section 6.5), together with a further ablation of the decoder’s pseudorandom seed-offset variable across five variants (optimised; three independently tested fixed constants; a K=3-realisation average), showing optimising σ is statistically indistinguishable from the two better-performing fixed values but significantly better than the worst one, while the fixed values themselves differ significantly from *each other* (Section 6.6), and a multi-instance validation across four additional real-city benchmarks (three subsets and one N=200 scale-up, all now at 10 seeds), showing MOEA/D last on every instance but SMS-EMOA’s and RVEA’s ranks otherwise varying by instance and the NSGA-II/NSGA-III ordering reversing, without significance, on two of the four, under a joint Holm correction across the four instances (Section 6.7), and a directional cross-check of the oracle’s fibre transmittance-vs-distance law against SeQUeNCe, an independent discrete-event quantum network simulator (Spearman ρ=1.0 across seven distances; Section 7, Threats to validity).
5. A public reproducibility package (QI-Bench-Pareto-v1) containing the pooled evolutionary non-dominated front (union of all seven benchmark algorithms, NSGA-II, NSGA-III, MOEA/D, SMS-EMOA, RVEA, SPEA2 and AGE-MOEA-II: 561 unique objective vectors, corresponding to 944 structurally distinct decoded topologies, with full per-point algorithm/seed provenance), the analytical oracle, and the experimental scripts.

## 2. Related Work

### 2.1. Quantum Network Design

The Quantum Internet roadmap of Wehner et al. [1] identifies six stages from trusted-node QKD to fully fault-tolerant entanglement distribution; complementary visions are due to Kimble [2], Cacciapuoti et al. [4] and Pirandola et al. [19]. The physical building block of any stage beyond stage two is the quantum repeater, originally proposed by Briegel, Dür, Cirac and Zoller (BDCZ) [9] and refined by subsequent generations of memory-based and all-photonic protocols [10,11,20]. More recent repeater-protocol work targets the same fidelity–rate trade-off from the physical-layer side that this paper takes as given: Ghosal et al. [21] optimise teleportation fidelity under a minimal-entanglement budget, Bayrakci and Ozaydin [22] use the quantum Zeno effect to suppress repeater decoherence, and Fuentealba et al. [23] apply machine learning to make repeater chains robust to component failure. These protocol-level advances are complementary to, not competing with, the topology-design problem addressed here: any of them could replace the BDCZ link model inside the oracle of Section 4.2 without changing the optimisation formulation itself. The Micius satellite [24,25] demonstrated intercontinental entanglement; subsequent missions have explored daylight QKD [26] and continental deployments [27,28]. Fibre-based QKD has been deployed over 400 km terrestrially [29] and twin-field protocols extend the reach further [30,31]. End-to-end fidelity over multi-hop chains follows the Werner-state concatenation rule; the bottleneck rate scales with detector efficiencies, source rates and swap-success probabilities. We adopt these models in the analytical oracle of Section 4.2.

### 2.2. Topology Optimisation for Quantum Networks

Dai et al. [14] optimise entanglement-distribution rate and repeater placement along a chain under end-to-end fidelity constraints. Related earlier formulations treat the network as given and analyse how it is used rather than how it should be built: Schoute et al. [32] study routing shortcuts on quantum networks, and Vardoyan et al. [33] give a stochastic analysis of a single entanglement-distribution switch. Genetic optimisation has also been applied to the different (inverse) problem of inferring an existing quantum network’s topology from indirect measurements [15], rather than designing one. Jallow and Khan [34] propose deep Q-network routing within fixed topologies; related RL approaches have been explored for routing under decoherence [35] and for entanglement distribution [36]. The RELiQ framework [16] combines a graph neural network with deep reinforcement learning for routing, again on fixed topologies. None of these works addresses *many-objective* topology design at intercontinental scale.

### 2.3. Recent Quantum-Network Optimisation (2024–2025)

Closer to the present work, several very recent papers optimise quantum-repeater networks with gradient- or surrogate-based methods rather than evolutionary search. Avis and Krastanov [37] use stochastic automatic differentiation to optimise rate–fidelity trade-offs and repeater placement in a 2-D plane, but for a single scalarised objective and small networks (up to four end nodes), not a many-objective Pareto front at intercontinental scale. Prielinger et al. [38] train machine learning surrogates to accelerate single-objective optimisation of quantum memory allocation and protocol parameters, a similar surrogate motivation to our companion paper [18] but applied to parameter tuning within a fixed topology rather than to topology design itself. Rabbie et al. [39] design quantum networks constrained to existing classical infrastructure, a complementary constraint we do not impose here. Pouryousef et al. [40] propose an overlay network for entanglement distribution over a fixed physical topology. Liu et al. [41] jointly design topology and resource allocation for entanglement distribution, a single-objective formulation solved by heuristic search rather than many-objective evolutionary optimisation. More broadly, Helsen and Wehner [42] propose a randomised-benchmarking procedure for individual quantum network links; standardised benchmarks at the level of full topology-design search, which this paper’s released Pareto front and oracle aim to help provide, remain comparatively scarce. None of these works performs many-objective evolutionary topology design over a heterogeneous, intercontinental candidate space; accordingly, we state our contribution precisely as the first such framework, rather than as topology design for quantum networks in general, which several of the works above already address under narrower (single-objective or fixed-topology) formulations.

### 2.4. Many-Objective Evolutionary Algorithms

NSGA-III [17] extends NSGA-II [43] to four or more objectives via reference-point-based niching [44]; alternative many-objective approaches include θ-DEA [45], KnEA [46] and indicator-based selection schemes [47,48]. MOEA/D [49] decomposes the multi-objective problem into scalar subproblems; variants include MOEA/D-DRA [50] and MOEA/D with adaptive weight adjustment [51]. We use the canonical implementations from pymoo [52]. Standard performance indicators include hypervolume (HV) [53,54], inverted generational distance (IGD) [55,56], spacing (Schott’s [57] canonical Manhattan-distance definition, used here precisely as specified in Section 5) and cardinality of the deduplicated front. Li et al. [58] survey how such diversity indicators behave specifically in the many-objective regime, which motivates our reporting spacing alongside HV and IGD rather than in place of them.

### 2.5. Surrogate-Assisted Approaches

A companion paper [18] introduces the QI-Bench-100 benchmark and a heterogeneous graph neural network surrogate for topology evaluation, building on graph neural network foundations [59,60,61] and their application to network performance prediction [62,63]. That companion paper reports that, once feature preparation is included, the trained surrogate is not faster than the analytical oracle except at large GPU batch sizes, and even then only modestly (∼13%, amortising over roughly 50,000 evaluations); it is presented there as a systematic multi-baseline comparison rather than a demonstrated wall-clock speed advantage. In the present work, the analytical oracle is used directly: the 11-dimensional mixed representation admits dense Pareto fronts in only ∼ 8000 evaluations per run, well below the batch size at which a learned surrogate would be expected to pay off.

## 3. Problem Formulation

We model a candidate global quantum network as a heterogeneous undirected graph G=(V,E) with N=|V|=100 cities distributed across five regions (Europe 30, North America 20, Asia-Pacific 25, Middle East/Africa 15, South America 10) and $E=E_{f}\cup E_{s}\cup E_{g}$ partitioned into fibre, satellite and free-space-optical (FSO) edge sets. Cities are a fixed, hard-coded set of national/financial capitals, major population centres, and (for the North American and European subsets specifically) several quantum-research hub cities (e.g., Oak Ridge, Pasadena, Princeton, Delft, Innsbruck); the per-region counts above are a deliberate allocation rather than a proportional sample of world population, favouring regions with denser existing or planned quantum-networking infrastructure and research activity. Full city names, countries and coordinates are given in cities.csv (released with the code).

### 3.1. Per-Link Physics

For an edge e=(u,v) of geodesic distance $d_{uv}$ and medium m∈{f,s,g}, the analytical oracle computes a triplet $\left(F_{uv},R_{uv},L_{uv}\right)$ for fidelity, rate and latency. Fibre links use a BDCZ chain of repeaters of maximum length $L_{\mathrm{rep}}=50$ km with attenuation α=0.20 dB/km, detector efficiency η=0.85, base fidelity $F_{0}=0.99$ and Bell-state-measurement swap success $p_{\mathrm{swap}}=0.50$. Satellite links follow a downlink–downlink geometry with LEO altitude 500 km, 30 cm transmit and 1 m receive apertures, atmospheric transmittance 0.80, pointing loss 3 dB, base fidelity $F_{0}^{s}=0.83$ (Micius-calibrated [24]) decaying with great-circle ground separation *d* (not slant range; only the geometric coupling efficiency, which sets the rate, depends on slant range—see Equation (A9) and Appendix A.2) at $\gamma_{s}=5\times 10^{-6}$ km^−1^, detector efficiency $\eta_{s}=0.50$ and source rate $r_{s}=5.9\times 10^{6}$ Hz. FSO links use extinction 0.50 dB/km up to a maximum distance of 150 km, base fidelity $F_{0}^{g}=0.92$ decaying at $\gamma_{g}=10^{-4}$ km^−1^, detector efficiency $\eta_{g}=0.70$ and source rate $r_{g}=10^{7}$ Hz. The full set of parameters is reproduced in Table 1.

### 3.2. End-to-End Objectives

For a source–destination pair (s,t) we select the path $\pi_{st}$ that maximises the exact end-to-end Werner-composed fidelity, via a maximum Werner-composed-fidelity path search. (A Dijkstra-like relaxation; not a classical widest/bottleneck path, and not the −logF shortest-path proxy, which is *not* optimality-preserving. In the Werner parameter, the problem does admit an exact additive form; Section 4.2 gives it and the resulting optimality argument.) The four end-to-end quantities are then aggregated as means over the subset P⊆V2 of city pairs for which *some* path exists in the candidate topology (i.e., the pair is connected at all); unlike the coverage indicator $C_{\tau }$ below, this aggregation set is not additionally filtered by a fidelity threshold, so $\bar{F}$, ${\bar{R}}_{\log}$ and $\bar{L}$ are means over every connected pair regardless of how low $F_{st}$ falls for that pair. Rate is aggregated in log space per pair *before* averaging (i.e., ${\bar{R}}_{\log}={\mathrm{mean}}_{t}\left[\log\left(1+R_{st}\right)\right]$, not $\log(1+{\mathrm{mean}}_{t}\left[R_{st}\right])$—these differ by Jensen’s inequality, and only the former matches both this paper’s and the companion paper’s oracle implementation):

(1) $$\begin{array}{cc} \bar{F} & =\frac{1}{\left|P\right|}\sum_{(s,t)\in P}F_{st}, \end{array}$$

(2) $$\begin{array}{cc} {\bar{R}}_{\log} & =\frac{1}{\left|P\right|}\sum_{(s,t)\in P}\log\left(1+R_{st}\right), \end{array}$$

(3) $$\begin{array}{cc} \bar{L} & =\frac{1}{\left|P\right|}\sum_{(s,t)\in P}L_{st}, \end{array}$$

(4) Cτ=1N2{(s,t):Fst≥0.70}.

The end-to-end fidelity along path $\pi_{st}=v_{0}v_{1}\ldots v_{h}$ follows the Werner concatenation $F_{st}=\frac{1}{4}+\frac{3}{4}\prod_{i=1}^{h}\frac{4F_{v_{i-1}v_{i}}-1}{3}$. This is not an independent modelling choice: writing each link’s Werner parameter as $w_{e}=\left(4F_{e}-1\right)/3$, repeated application of the entanglement-swapping composition rule (Equation (6), derived in Section 4.2) collapses to the product $w_{v_{0}v_{h}}=\prod_{i}w_{v_{i-1}v_{i}}$, and substituting back $F=\frac{1}{4}+\frac{3}{4}w$ gives exactly the expression above; it is the two-party BDCZ swap composed h−1 times along the path, not a separate approximation. The rate is the bottleneck $R_{st}=\min_{i}R_{v_{i-1}v_{i}}\cdot p_{\mathrm{swap}}^{h-1}$; the latency is $L_{st}=\sum_{i}L_{v_{i-1}v_{i}}$. The 0.70 fidelity threshold in Equation (4) is a design choice rather than a physical constant, so we tested its sensitivity by re-decoding every point on the pooled evolutionary front (Section 6.2) from its stored parameter vector and recomputing $C_{\tau }$ at four alternative thresholds (0.50, 0.60, 0.80, 0.90) using the same exact path search, not a proxy (Script: coverage_threshold_sensitivity_v3.py, released with the reproducibility package). The resulting per-topology coverage ranking is essentially unchanged at 0.50, 0.60 and 0.80 (Spearman ρ≥0.99 against the canonical 0.70 threshold across all 561 points), so $C_{\tau }$’s role in the correlation structure and effective-dimensionality result (Section 7) is not an artefact of this specific threshold value. At 0.90, the ranking correlation drops to ρ=0.20, but this reflects a floor effect, not ranking instability: mean $C_{\tau }$ collapses to 0.006 at that threshold (almost no city pair reaches 90% end-to-end fidelity on this benchmark, so nearly every topology reports a coverage near zero, leaving little genuine variation left to rank).

### 3.3. Optimisation Problem

The many-objective topology design problem is

(5) $$\min_{G\in G}\;\left(-\bar{F}\left(G\right),\;-{\bar{R}}_{\log}\left(G\right),\;\bar{L}\left(G\right),\;-C_{\tau }\left(G\right)\right),$$

where G denotes the space of admissible topologies. Direct optimisation over G is intractable at either of its two natural encodings: a binary edge mask that only decides edge/no-edge and assumes a single implicit medium ($\left|G\right|=2^{4950}$), or the more expressive, genuinely free per-edge categorical choice among {no edge, fibre, satellite, FSO} that Section 6.3 benchmarks directly ($\left|G\right|=4^{4950}$ under one medium per city pair). We introduce an eleven-variable mixed representation in Section 4.1 that avoids searching either space directly.

## 4. Methodology

### 4.1. Eleven-Variable Mixed Parametric Representation

We define a deterministic decoder Φ:X→G, $X=\prod_{i=1}^{11}\left[{\mathrm{XL}}_{i},{\mathrm{XU}}_{i}\right]\subset R^{11}$, that maps a parameter vector x∈X into a heterogeneous topology. Ten of the eleven variables are physical design parameters (per-medium neighbour budgets, ranges, inclusion probabilities and the inter-region bonus); the eleventh, σ, is a pseudorandom seed offset rather than a design choice (Section 6.6 tests and discusses this distinction directly). Four of the eleven variables ($k_{f}$, $k_{s}$, $k_{g}$, σ) are deterministically discretised to the nearest integer before decoding; the remaining seven are used as real-valued inputs directly. We therefore describe this as a mixed, rather than purely continuous, representation. The eleven variables and their ranges are listed in Table 2. They cover *k*-nearest-neighbour budgets per medium, maximum geodesic distances per medium, per-medium inclusion probabilities, an inter-region bonus that increases the probability of trans-continental edges (applied to fibre and satellite candidates only, not FSO—see Algorithm 1), and a seed offset that initialises the random-number generator governing every Bernoulli edge-inclusion draw in the decoder, not only tie-breaking.

The decoder uses exactly the eleven variables listed above—no additional parameters are computed or referenced anywhere in the edge-sampling logic. The decoder is given as Algorithm 1, transcribed directly from the released implementation rather than as an idealised summary.

#### 4.1.1. Why |E|<5

The threshold is a minimal-connectivity guard, not a tuned hyperparameter. On a 100-node instance, a graph with fewer than 5 edges cannot connect more than 10 nodes even in the best case (a forest has at most |V|−1 edges spanning |V| nodes, so 4 edges span at most 5 disjoint components of 2 nodes each), leaving P near-empty and every downstream objective (Equations (1)–(4)) either undefined or computed over a handful of pairs unrepresentative of the topology the decoder was asked to produce. Rather than let such vectors compete on whatever few pairs they happen to connect, the decoder rejects them outright with a fixed, uniformly dominated penalty, so the search never has to disentangle “genuinely good on few pairs” from “degenerate.” The exact cut-off is intentionally conservative (most decoded topologies well exceed it): it only needs to catch the pathological, near-empty cases, and 5 was chosen as the smallest value for which this near-empty regime is already guaranteed under the argument above, not fitted to any observed result.
**Algorithm 1** Topology decoder Φ(x).**Require:** parameter vector $x\in X\subset R^{11}$**Ensure:** topology G=(V,E)  1:$k_{f}\leftarrow \max\left(1,\mathrm{round}\left(x_{1}\right)\right)$; $k_{s}\leftarrow \max\left(1,\mathrm{round}\left(x_{2}\right)\right)$; $k_{g}\leftarrow \max\left(0,\mathrm{round}\left(x_{3}\right)\right)$  2:$\sigma \leftarrow \mathrm{round}(x_{11})\;\mathrm{mod}\;1000$;   initialise RNG with seed σ  3:E←∅       ▹ a *set* of canonical triples (min(u,v),max(u,v),medium): a pair drawn from both endpoints is stored once, but is drawn twice (see text)  4:**for** each node u∈V **do**                              ▹ **Fibre**  5:      $N_{f}^{u}\leftarrow \left\{v\neq u:d_{uv}\leq d_{f}^{\max}\right\}$, sorted ascending by $d_{uv}$, top-$k_{f}$  6:      **for** $v\in N_{f}^{u}$ **do**  7:            $p\leftarrow p_{f}$; **if** region(u)≠region(v) **then** $p\leftarrow p\cdot (1+\beta_{r})$  8:            sample b∼Bernoulli(min(p,1)); **if** b=1 **then** E←E∪{(u,v,f)}  9:      **end for**              ▹**Satellite**—note the 500 km floor, independent of $d_{s}^{\max}$10:      $N_{s}^{u}\leftarrow \left\{v\neq u:500\leq d_{uv}\leq d_{s}^{\max}\right\}$, sorted ascending by $d_{uv}$, top-$k_{s}$11:      **for** $v\in N_{s}^{u}$ **do**12:            $p\leftarrow p_{s}$; **if** region(u)≠region(v) **then** $p\leftarrow \min(1,p\cdot \left(1+\beta_{r}\right))$13:            sample b∼Bernoulli(p); **if** b=1 **then** E←E∪{(u,v,s)}14:       **end for**                   ▹ **FSO**—no inter-region bonus applied15:       **if** $k_{g}>0$ **then**16:           $N_{g}^{u}\leftarrow \left\{v\neq u:d_{uv}\leq d_{g}^{\max}\right\}$, sorted ascending by $d_{uv}$, top-$k_{g}$17:           **for** $v\in N_{g}^{u}$ **do**18:                sample $b∼\mathrm{Bernoulli}(p_{g})$; **if** b=1 **then** E←E∪{(u,v,g)}19:           **end for**20:       **end if**21:**end for**22:**if**|E|<5**then return**None (topology rejected; oracle returns the fixed penalty vector $\left(\bar{F},{\bar{R}}_{\log},\bar{L},C_{\tau }\right)=\left(0.5,\;0,\;1.0,\;0\right)$, dominated by every topology reachable in practice)23:**end if**24:**return**G=(V,E)

#### 4.1.2. Effective Per-Pair Edge Probability

The outer loop visits every node, so an unordered pair {u,v} that lies in *both* endpoints’ top-*k* lists is visited twice and receives two independent Bernoulli draws, while the canonical set *E* stores it at most once. Its effective inclusion probability is therefore $1-{\left(1-p\right)}^{2}$, not *p*; a pair that appears in only one endpoint’s list keeps probability *p*. This is a property of the decoder as defined and used for every result in this paper, not an artefact of the pseudocode, and it is substantial rather than marginal: on the 100-city instance, 48.7–55.4% of candidate fibre pairs are mutual *k*-nearest neighbours across the $\left(k_{f},d_{f}^{\max}\right)$ range the search explores. A Monte-Carlo check against the implementation reproduces both probabilities exactly (Appendix A.5). The consequence for interpretation is that $p_{f}$, $p_{s}$ and $p_{g}$ are edge-*proposal* probabilities per endpoint visit rather than per-pair inclusion probabilities; the decoder remains a well-defined deterministic function of (x,σ), and no result changes, but the mapping from *p* to expected density is the one stated here.

### 4.2. Analytical Oracle: Exact Werner-Optimal Path Search

The oracle is derived from the analytical BDCZ oracle used to generate the companion QI-Bench-100 benchmark [18], with three corrections identified by post hoc physics audits of this paper’s own oracle and applied to every result reported here.

*(a) Satellite line of sight.* The satellite model placed a source at h=500 km over the midpoint and computed the slant range as $\sqrt{{\left(d/2\right)}^{2}+h^{2}}$, a flat-Earth expression, with the decoder free to request satellite links out to 15,000 km and no visibility constraint at all. A single satellite at that altitude can see both ground stations only up to a great-circle separation of 2Rarccos(R/(R+h))=4891 km (Equation (A6)); beyond it, the Earth is in the way. Auditing the previously released front against this constraint found that 14.6% of all satellite edges (69,800 of 476,827, spanning 382 distinct city pairs) were links that cannot physically exist, in a front whose pooled edge count was 87.9% satellite. We replace the slant range with the spherical expression of Equation (A5), declare links beyond the visibility limit infeasible, and cap the decoder’s own bound at that limit so the search cannot propose them.

*(b) Two-arm coincidence efficiency.* The satellite link budget squared the atmospheric transmittance while applying the geometric, pointing and detector efficiencies once, which is inconsistent under either reading of those terms. Heralding a pair requires a detection at both stations, so all per-arm factors are now squared (Equation (A8)), and the delivery latency is one transit time rather than the sum of both arms’.

*(c) Fibre latency and the rate floor.* The companion benchmark’s fibre-link latency formula scaled quadratically rather than linearly with distance, which we fix to the linear form implied by summing per-span round-trip propagation delays; and the per-hop rate floor of $10^{-3}$ s^−1^, which served no numerical purpose, is removed (Appendix A).

All results in this paper were recomputed from scratch under the corrected oracle. The companion benchmark’s own published results predate these corrections and are not revised here, so the two papers’ absolute numbers are not directly comparable version-for-version, though the underlying physical models are otherwise the same. Link-level fidelity, rate and latency are precomputed once per medium pair ($O(N^{2})$). When more than one medium connects the same city pair, only the higher-fidelity medium is retained for path search (the lower-fidelity parallel edge remains part of the stored topology but cannot itself be selected).

#### Why the Search Is Exact

A Werner state with fidelity *F* to the target Bell pair is a mixture of that Bell pair with weight *F* and the maximally mixed two-qubit state with weight 1−F; equivalently, in the Bell basis, it carries weight w=(4F−1)/3∈[0,1] on the target Bell pair beyond the uniform background and weight (1−w)/4 on each of the four Bell states. BDCZ entanglement swapping performs a Bell-state measurement at the intermediate node on two independently prepared Werner links with parameters $w_{1}$, $w_{2}$; because a Bell measurement acting on a product of two such mixtures maps a matching pair of off-diagonal weights to a single output weight $w_{1}w_{2}$ and every other combination to the (twirled-away) background, the output state is itself Werner with parameter $w_{12}=w_{1}w_{2}$—the standard result for concatenating Werner-diagonal states under BDCZ swapping [9,64]. Writing $F_{e}=\left(3w_{e}+1\right)/4$ turns $w_{12}=w_{1}w_{2}$ into the equivalent fidelity form used throughout this paper,

(6) $$F_{12}=F_{1}F_{2}+\frac{1}{3}\left(1-F_{1}\right)\left(1-F_{2}\right).$$

Iterating this two-link composition along a path of *h* hops gives

(7) $$w_{12\cdots h}=\prod_{i=1}^{h}w_{v_{i-1}v_{i}},\;\mathrm{hence}\;F_{st}=\frac{1}{4}+\frac{3}{4}\prod_{i}w_{v_{i-1}v_{i}},$$

so the composition is an exact *product* in *w* (this identity is algebraic, not an approximation; we verify it numerically to machine precision) (werner_path_validation_v3.py and werner_path_validation_v3.json: maximum absolute error $2.2\times 10^{-16}$ over $2\times 10^{5}$ random pairs). Two consequences follow. First, because $F_{st}$ is a strictly increasing function of $\prod_{i}w_{e}$ and $0<w_{e}\leq 1$ on every usable link, path fidelity is non-increasing in additional hops, every sub-path of a maximum-∏w path is itself maximal between its endpoints, and the greedy Dijkstra-like relaxation on *F* is therefore optimal by the standard optimal-substructure argument for multiplicative shortest paths with weights in (0,1]. Second, taking logarithms turns Equation (7) into an *exact* additive shortest-path problem with non-negative edge weights −$\log w_{e}$. What is *not* exact is the −$\log F_{e}$ proxy: *F* is not multiplicative under swapping, so minimising $\sum_{i}-$$\log F_{v_{i-1}v_{i}}$ selects a Werner-suboptimal path for 17.8% of reachable pairs on this benchmark’s own topologies—not a negligible fraction, which is why this paper does not use it.

Beyond the substitution identity above, the implementation is validated two further, independent ways against exhaustive and alternative-algorithm ground truths; Appendix A.5 gives both checks in full.

For each source node, the oracle runs one such search, for a total cost of O(N) Dijkstra-like searches, each O(|E|logN), run in pure Python v3.11.15; this is slower in wall-clock terms than a single vectorised $O(N^{3})$ floyd_warshall call over the −logF proxy cost matrix, but is used throughout this paper so that every reported result is generated by a single oracle implementation and physical parameter set (Section 5 reports the exact measured per-call cost of this exact search). Appendix A gives the complete link-level model from which the $F_{e}$, rates and latencies are computed.

### 4.3. Evolutionary Algorithm Configuration

We instantiate NSGA-II [43], NSGA-III [17], MOEA/D [49], SMS-EMOA [48] and RVEA [65] from pymoo [52] with simulated binary crossover (SBX) [66] and polynomial mutation (PM) [67]: SBX (η=15, $p_{c}=0.9$), PM (η=20, $p_{m}=1/n_{\mathrm{var}}$), and Latin-hypercube sampling initialisation, identically across all seven algorithms, with no per-algorithm operator or hyperparameter tuning (Threats to validity, item ii). NSGA-II, SMS-EMOA and RVEA take an explicit population size and use 104 over 80 generations (8320 oracle calls per run); NSGA-III uses the same 104 population size, set independently of its reference-direction count. NSGA-III, MOEA/D and RVEA additionally require das-dennis reference directions with $n_{\mathrm{partitions}}=6$, which for four objectives yield 4+6−16=84 directions (verified directly against pymoo), not 104—unlike NSGA-III and RVEA, whose population sizes are set independently of their reference-direction count (verified for RVEA by direct calibration against this oracle: exactly 104 evaluations per generation with zero intercept, despite only 84 reference directions), MOEA/D’s population size in pymoo *is* its number of reference directions, so running MOEA/D at the same 80 generations as the other algorithms would give it only 6720 oracle calls, an unintended ∼19% smaller evaluation budget. We instead give MOEA/D 99 generations (84×99=8316 evaluations), matching the other six algorithms’ (population 104) 8320 to within 0.05%, and report the actual measured oracle-call count for every run (not an assumed one) alongside every result. Total budget per run: ∼$8.3\times 10^{3}$ oracle calls for all seven algorithms.

## 5. Experimental Setup

### 5.1. Software and Hardware

We use Python v3.11.15 with pymoo v0.6.1.6 for the evolutionary algorithms, NumPy v2.4.3 and SciPy v1.17.1 for the oracle, on an AMD Ryzen 9 7900X CPU (12 cores/24 threads, single-threaded per run). No GPU is required.

### 5.2. Baselines

We compare NSGA-III against NSGA-II [43] (same operators, no reference directions, fast non-dominated sorting + crowding), MOEA/D [49] (Tchebycheff scalarisation, $n_{\mathrm{neighbors}}=15$, same reference directions as NSGA-III), SMS-EMOA [48] (hypervolume-contribution-based survival selection, a natural comparison given that HV is this paper’s primary indicator), and RVEA [65] (reference-vector-adaptation, a more recent many-objective algorithm), plus SPEA2 [68] (archive-and-density selection) and AGE-MOEA-II [69] (2022, adaptive geometry estimation instead of a fixed reference-direction set). All seven use the same decision-variable bounds and an approximately matched objective-function evaluation budget, not literally the same population size and generation count: NSGA-II, NSGA-III, SMS-EMOA, RVEA, SPEA2 and AGE-MOEA-II all use population 104 over 80 generations (8320 oracle evaluations); MOEA/D’s population in pymoo is architecturally tied to its number of reference directions (84, Section 4.3), so it instead runs 99 generations (8316 evaluations) to match the same budget rather than being forced onto an artificially small population for the same generation count.

### 5.3. Performance Indicators

We report (a) *hypervolume* (HV) with reference point r=[1.1,1.1,1.1,1.1] in normalised objective space (chosen 10% beyond the empirical nadir so all Pareto points contribute positively to the HV; we recompute HV at 1.05 and 1.20 as a sensitivity check directly from the cached per-run fronts and find the ranking unchanged at both alternative reference points, with per-seed Spearman rank correlation against the 1.10 ranking of 0.983 and 0.987 respectively), (b) *inverted generational distance* (IGD) computed against the non-dominated union of all final fronts within each experimental block, and (c) *spacing* as the standard deviation of nearest-neighbour Manhattan ($L_{1}$) distances in normalised objective space, following Schott’s (1995) canonical definition [57]. This paper reports two *primary* blocks—the algorithm comparison (NSGA-II, NSGA-III, MOEA/D, SMS-EMOA, RVEA, SPEA2, AGE-MOEA-II, 30 seeds each, Section 6.1) and the decoder ablation (eight variants, 30 seeds each, Section 6.5)—plus several further blocks each with their own block-specific normalisation, scoped to a narrower question: the sigma ablation (Section 6.6), the categorical baselines under both SBX/PM and category-native operators (Section 6.3), the shared-scale master comparison of decoder vs. both categorical baselines (Section 6.3), random search vs. the decoder, and the four-instance multi-instance validation (Section 6.7). Every block uses its own min–max normalisation computed from the Pareto fronts within that block, so HV/IGD values are comparable *within* a block but *not across* blocks unless a shared reference is explicitly constructed, as the master comparison does. We deliberately do not report a third, separately normalised single-algorithm per-seed table within the two primary blocks: because such a table would use a different reference front from the algorithm-comparison block for nominally the same runs, its HV values would not be comparable to the algorithm-comparison table despite superficially looking like they should be; per-seed values for every algorithm are instead available directly in the released JSON results. Each block’s reference front is deduplicated by objective vector before use (Section 6.1 below). The algorithm-comparison block’s reference front is built from all seven algorithms’ raw per-seed fronts and contains 561 points. This is the same object as the released pooled evolutionary front used throughout Section 6.2 and everywhere else in this paper: SPEA2 and AGE-MOEA-II were added to the algorithm comparison after a pooled front unioning only the five original benchmark algorithms (513 points) had already been built, which briefly left that front and the 561-point comparison reference out of step; every downstream quantity (connectivity, PCA, correlations, threshold and routing sensitivity, classical-baseline dominance, the representative topology maps, and the released package) is now recomputed against the unified 561-point, seven-algorithm front. Applying the identical recipe to the five original algorithms alone reproduces the 513-point front exactly, so the two differ only by the two added algorithms; the difference is not a simple addition, however, because their points both enter the reference and dominate some points that were non-dominated among the five. Of the 561 points, 315 are attained only by the original five, 215 only by SPEA2 or AGE-MOEA-II, and 31 by both groups. Every HV, IGD and spacing value in Section 6.1, and every quantity elsewhere in the paper computed from the pooled evolutionary front, uses this same 561-point reference. The ablation block’s primary reference front (the igd field in the released results) is built only from the full decoder’s (variant A) pooled, deduplicated fronts across its 30 seeds (358 points), not from all eight variants jointly, so that every variant’s IGD measures its distance from the unablated decoder specifically rather than from an average across variants that already differ by construction; this necessarily makes variant A’s own IGD close to zero by construction, which is not a symmetric comparison across variants. We therefore additionally report igd_union: a second, genuinely symmetric IGD computed for every variant (including A) against the shared, deduplicated union of all eight variants’ pooled fronts: a symmetric shared reference to which every variant contributes, so that no variant is scored against a reference built exclusively from its own results (the union does contain each variant’s own points, by construction) (Section 6.5).

### 5.4. Statistics

The algorithm comparison uses 30 independent seeds (42–71). The decoder ablation uses 30 seeds (42–71). We report mean ± sample standard deviation (ddof=1).

## 6. Results

### 6.1. Algorithm Comparison

**Figure 1:**
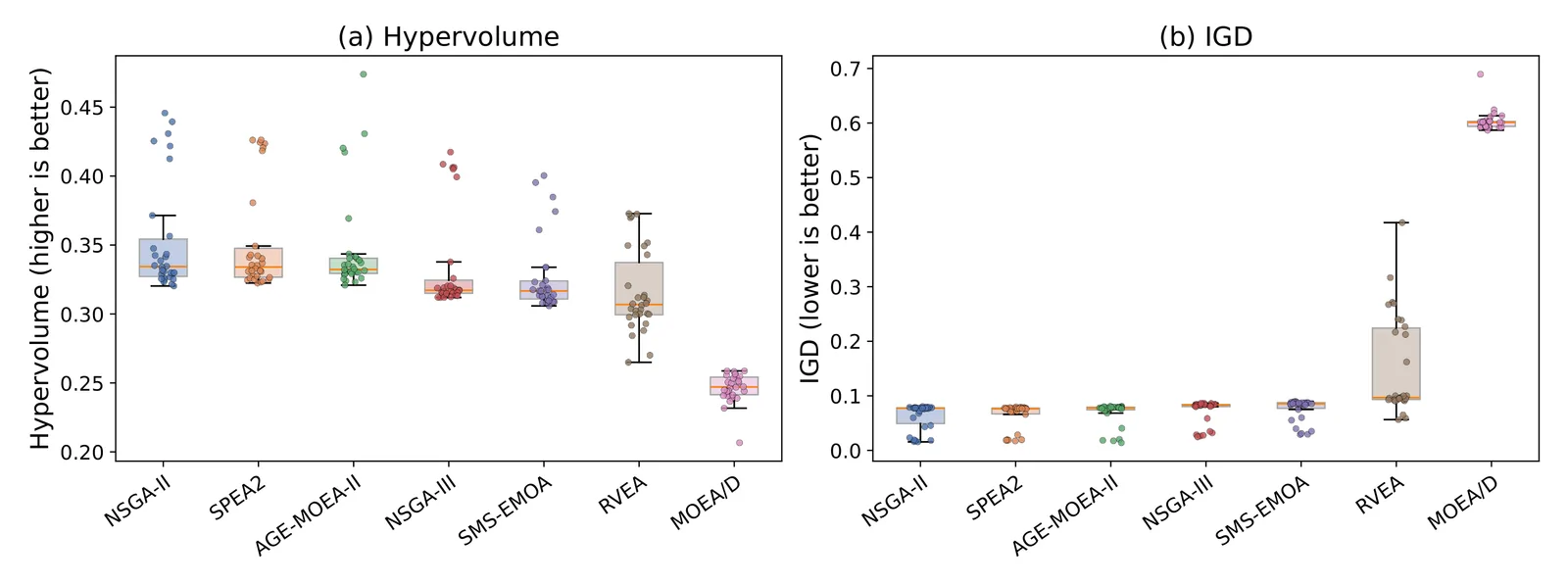
Algorithm comparison: hypervolume (**left**, higher is better) and IGD (**right**, lower is better) for all seven algorithms over 30 independent seeds (canonical oracle), ordered by mean HV and scored against the single shared reference front of Table 3. Boxes show the median and interquartile range; individual seeds are overlaid as jittered points so that any single outlier run is visible directly rather than folded into a mean ± std bar. The bimodality of NSGA-II’s and NSGA-III’s per-seed HV (Section 6.1) is visible as two point clusters.

Table 3 reports HV, IGD, spacing, $|F^{★}|$ and runtime for all seven algorithms over 30 seeds. Figure 1 visualises the comparison. The seven span 2001–2022 and cover the dominance-and-density (NSGA-II, SPEA2), reference-direction (NSGA-III, RVEA, MOEA/D), indicator (SMS-EMOA) and adaptive-geometry (AGE-MOEA-II) families. All are run under identical conditions—same decoder, oracle, bounds, SBX/PM operators, LHS sampling, 8320-evaluation budget and 30 seeds—and all seven are scored against a *single* reference front and a single min–max normalisation built once from the union of all 210 raw per-seed fronts (Script and results: unified_seven_algorithm_comparison_v3.py and the corresponding .json). So every HV, IGD and spacing value in this section lies on exactly the same scale. This matters: scoring a subset of algorithms against a bounding box built from a different subset produces numbers that look comparable and are not, and it is the reason we re-score rather than quote the five- and two-algorithm blocks side by side.

SPEA2’s $|F^{★}|=103.8\pm 0.9$ is not far from a rounding artefact: its archive returns close to a full population of 104 mutually non-dominated, duplicate-free objective vectors in every one of the 30 runs.

Under Holm-corrected independent-samples testing across the full family of 63 tests, 49 are significant, with mostly large effect sizes where they are (|δ|≥0.50, most ≥0.60). The structure of the result is a *top group* that is clearly separated from the rest, not a single winner within it. NSGA-II, SPEA2 and AGE-MOEA-II are mutually indistinguishable on all three metrics ($p_{\mathrm{Holm}}=0.215$–1.000, |δ|≤0.22 throughout) and each is significantly ahead of NSGA-III, SMS-EMOA, RVEA and MOEA/D on HV. NSGA-III is significantly behind the top three on HV and IGD ($p_{\mathrm{Holm}}\leq 0.002$ throughout, effect sizes δ≈0.60–0.63 in their favour). Against SMS-EMOA, NSGA-III is statistically indistinguishable on HV and IGD ($p_{\mathrm{Holm}}=1.000$ and 0.085) but significantly *better* on spacing under this primary design ($p_{\mathrm{Holm}}=0.009$, δ=−0.52 favouring NSGA-III, since spacing is minimised); the paired sensitivity design does not confirm this spacing difference ($p_{\mathrm{Holm}}=0.345$), so we read the two as indistinguishable in HV/IGD and unresolved on spacing rather than ranked. MOEA/D is last on HV and IGD by a wide margin under every comparison.

Two points are worth stating explicitly, because the same design that produced this table produced a different-looking result before a satellite-geometry error in the oracle (Section 4.2) was corrected and every result recomputed. First, NSGA-II’s mean-HV lead over NSGA-III survives the full seven-algorithm Holm family on all three metrics ($p_{\mathrm{Holm}}=0.001$ on HV, <0.001 on IGD, 0.003 on spacing), so the top-group/NSGA-III separation is not an artefact of family size the way it was under the uncorrected oracle. Second, the two additions are still not a footnote to an NSGA-II result. AGE-MOEA-II, an adaptive-geometry method introduced in 2022, and SPEA2, a dominance-and-density method dating from 2001, both perform within noise of NSGA-II; AGE-MOEA-II reaches that level at roughly 60% of NSGA-II’s runtime. The most defensible ordering for this benchmark is therefore {NSGA-II, SPEA2, AGE-MOEA-II} > NSGA-III ≳ SMS-EMOA  > RVEA > MOEA/D: the first three form a tied group, and NSGA-III is separated from them but not cleanly from SMS-EMOA. This agrees with the Kruskal–Wallis omnibus result for spacing (H=131.450, $p<10^{-6}$, Table 4) and does not require treating same-seed-index runs across algorithms as matched pairs. The complete set of Holm-corrected pairwise independent-samples comparisons behind this ordering is reported in Table 5.

The mechanistic conclusion does not depend on which of the two statistical designs is preferred (Section 7 returns to this point). The front has low effective dimensionality, with PC1 explaining ≈96% of the variance, and the machinery built to counteract dominance resistance in higher-dimensional fronts—AGE-MOEA-II’s adaptive geometry estimation, NSGA-III’s reference-point niching, RVEA’s reference-vector adaptation, MOEA/D’s decomposition—produces no measurable gain over simple crowding-distance or archive-density selection within the top group; for NSGA-III, RVEA and MOEA/D, that machinery is accompanied by a substantial performance cost instead.

#### 6.1.1. Sanity Check: Does Evolution Beat Random Search?

An 11-dimensional mixed search space is small enough that much of the observed performance could come from the decoder’s own bounds and structure rather than from evolutionary search, since i.i.d. random sampling would exploit that structure almost as well. We tested this directly: 30 seeds of pure uniform random sampling over the same [XL,XU] bounds, same 8320-evaluation budget, same oracle, non-dominated-filtered at the end, re-scored against a reference front shared with NSGA-II’s own runs so the two are on the same HV scale (Script: random_search_baseline_v3.py, released with the reproducibility package; Scoring results: random_search_vs_decoder_shared_ref_v3.json). The shared reference used here belongs only to this comparison, so the HV values in this paragraph are *not* on the scale of Table 3: NSGA-II’s 0.443 here and its 0.353 there come from the same runs evaluated under different normalisations, and only within-paragraph comparisons are meaningful.

Against random search, NSGA-II obtains HV=0.443±0.054 rather than 0.407±0.021. The difference is statistically significant (Mann–Whitney $p=5.0\times 10^{-4}$, Cliff’s δ=+0.52), but its relative magnitude is modest, around 9%—smaller than one might expect for a purpose-built many-objective evolutionary algorithm against a naive baseline, and smaller still than the ∼21% gap observed under an earlier version of the oracle, where the uncorrected satellite geometry let the decoder’s own *k*-nearest-neighbour structure exploit more long-range edges than random sampling was likely to reach by chance.

The smaller, corrected gap reads as informative rather than damaging: much of the benefit already comes from the bounds and structure the 11-variable decoder itself imposes (Section 4.1), and evolutionary selection adds a genuine but comparatively modest further improvement on top of that—consistent with, not contradicting, this paper’s central claim that the representation itself, not any one algorithm’s search strategy, is what makes this problem tractable at all (Section 6.3 shows the same representation is necessary regardless of which optimiser is used on it).

#### 6.1.2. Common-Seed Paired Sensitivity Check

Each algorithm’s 30 runs also happen to share the same 30 RNG seed values (42–71), so a paired analysis blocking by seed index is also possible and is standard practice in the EC statistics literature for repeated-seed comparisons [70]. We report it here as a sensitivity check on the independent-samples design above, not as the primary claim, since pairing by seed index assumes seed *s* produces a comparable initial condition across algorithms with different population dynamics (MOEA/D’s decomposition, in particular, consumes its RNG stream differently from algorithms with different population and selection dynamics)—an assumption we do not need for the primary result. A Friedman omnibus test (Table 6) and Holm-corrected paired Wilcoxon signed-rank tests (Table 7) agree with the independent-samples design on 60 of the 63 comparisons (significant/not-significant verdict matching), with large matched-pairs rank-biserial effect sizes where significant ($|r_{rb}|\geq 0.63$). The three disagreements all involve NSGA-III at an already-borderline effect under the independent-samples design (Table 5): NSGA-III vs. RVEA on HV ($p_{\mathrm{Holm}}=0.013$ unpaired vs. 0.092 paired), and NSGA-III vs. MOEA/D and NSGA-III vs. SMS-EMOA on spacing. The two designs agree on NSGA-II vs. NSGA-III, and this time, they agree on a positive: both find it significant after the joint correction on all three metrics ($p_{\mathrm{Holm}}=0.001$/0.005 on HV, <0.001/0.020 on IGD, 0.003/0.023 on spacing, unpaired/paired), with a large effect size in NSGA-II’s favour under both (δ=+0.62, $r_{rb}=+0.74$ on HV). The top-group structure—{NSGA-II, SPEA2, AGE-MOEA-II} mutually tied and all clearly ahead of NSGA-III, RVEA and MOEA/D—is not in dispute under either design.

Spacing is computed after deduplicating each run’s front by objective vector (Section 6.1); without this step, MOEA/D’s raw non-dominated population routinely contains >80% exact duplicate objective vectors (verified directly), which mechanically deflates its nearest-neighbour distances and would read as a spurious spacing advantage rather than a genuine distributional one. We do not make a ranking claim on spacing among NSGA-II, NSGA-III and MOEA/D specifically. Between NSGA-III and SMS-EMOA, the primary (unpaired) design finds NSGA-III’s spacing significantly *better* ($p_{\mathrm{Holm}}=0.009$, δ=−0.52; spacing is minimised, so the negative sign favours the algorithm listed first, NSGA-III), but the paired design does not confirm this ($p_{\mathrm{Holm}}=0.345$, Table 7); we therefore regard their spacing comparison as design-dependent and unresolved rather than asserting a ranking. RVEA’s spacing remains markedly worse, and its much smaller unique-solution count below is consistent with that. MOEA/D’s much smaller unique-solution count (Table 3, $|F^{★}|=6.7$ vs. 89.9 and 65.1 for NSGA-II/NSGA-III) comes together with its lower hypervolume and substantially worse IGD, so no dimension of Table 3 supports reading MOEA/D as competitive with NSGA-II or NSGA-III on this problem once duplicates are correctly removed. SMS-EMOA and RVEA—included specifically to broaden the algorithm portfolio beyond dominance/decomposition-based methods—are no longer equally positioned: SMS-EMOA underperforms the top group (NSGA-II, SPEA2, AGE-MOEA-II) on all three metrics but is statistically indistinguishable from NSGA-III on HV and IGD, and the primary design finds it significantly *behind* NSGA-III on spacing (not confirmed by the paired design; Section 6.1 above), with a unique-solution count (81.5±6.0) closer to NSGA-II’s than to RVEA’s; RVEA, in contrast, still collapses to a very small unique-solution count (9.7±1.6) with markedly worse spacing, suggesting its reference-vector adaptation mechanism (tuned for many-objective problems with more than four objectives) does not find this specific four-objective, mixed-integer landscape especially favourable at a matched budget. SMS-EMOA’s improved standing relative to an earlier version of this oracle is consistent with its hypervolume-contribution selection mechanism rewarding solutions that push the front outward in whatever directions remain profitable—a mechanism that may have specifically benefited from the removal of the many now-infeasible, artificially attractive long-range satellite edges the uncorrected oracle offered; this is stated as a plausible explanation, not one we have tested directly.

#### 6.1.3. NSGA-II’s Mean Hypervolume Is Bimodal Across Seeds, Not Uniformly Elevated

Sorting NSGA-II’s 30 per-seed HV values reveals a clear gap: 24 seeds cluster in [0.320,0.371], while the remaining 6 seeds (43, 49, 54, 57, 63, 65) jump to [0.413,0.446]—a gap of 0.041 between the two clusters, larger than the spread within either one. The same pattern appears for NSGA-III (6 of its 30 seeds—43, 54, 56, 57, 63, 71—reach a similarly elevated cluster, [0.399,0.417] vs. [0.312,0.338] for the rest), with 4 of its 6 high seeds (43, 54, 57, 63) coinciding with NSGA-II’s own high-cluster seeds—a majority overlap rather than one or two shared values. This is not an artefact of a truncated run or a corrupted checkpoint: every seed used the identical, measured 8320-evaluation budget, and the high-HV runs also show markedly better IGD (0.016–0.023 vs. 0.043–0.081 for NSGA-II’s low cluster; 0.025–0.035 vs. 0.059–0.087 for NSGA-III), consistent with a genuinely different, better region of the search space rather than noise. We read this as evidence that the fitness landscape induced by this decoder and oracle is multimodal for reference/crowding-based selection (NSGA-II and, comparably here, NSGA-III), with a subset of LHS-sampled initial populations occasionally escaping to a substantially better basin. The overlap in *which* seeds escape is consistent with that basin being a property of the oracle/decoder landscape rather than of either algorithm’s selection mechanism, but we state this as an observation, not as an inference: both algorithms receive the same seed, the same population size (104) and the same LHS sampler, so seed *s* can produce identical or near-identical initial populations for the two, and the overlap may reflect shared starting points rather than a shared attractor. Distinguishing the two would require comparing the initial populations directly and measuring where the trajectories diverge, which we have not done. This is why we report non-parametric summaries alongside the mean below. For NSGA-II, both quartile ranks of the 30 sorted per-seed HV values (ranks 8 and 23 under the standard (n+1)/4 convention) fall inside the 24-seed low cluster, so the interquartile range is bounded entirely within it, IQR≤0.371−0.320=0.051, and does not itself span the gap to the high cluster; the same holds for NSGA-III’s 24-seed low cluster. A single IQR number is therefore not the most informative non-parametric summary here: because it sits entirely inside the majority cluster, it would read as an ordinary, unimodal spread and hide the very bimodality it is meant to surface. The two-cluster range breakdown reported above is the more diagnostic non-parametric description for this specific bimodal case, which is why we lead with it rather than a single median/IQR pair (the full mean/median/IQR breakdown for HV, IGD, spacing and runtime is additionally released with the reproducibility package for readers who want the conventional summary). This is also why Figure 1 shows the full per-seed distribution rather than only a mean-and-error-bar view. The practical implication is that NSGA-II’s mean-HV lead over NSGA-III should be read as “both algorithms reach a better basin on a similar, overlapping fifth of seeds, and NSGA-II is somewhat ahead in both regimes,” not as “NSGA-II is uniformly better every run”; median HV (0.334 for NSGA-II vs. 0.317 for NSGA-III) tells the more conservative, typical-run version of the same story. Identifying what specifically makes seeds 43/49/54/56/57/63/65/71 (the union of both algorithms’ high clusters) special (e.g., a property of the LHS initial sample, or of a downstream RNG draw in crossover/mutation) is left as future work.

#### 6.1.4. Convergence

**Figure 2:**
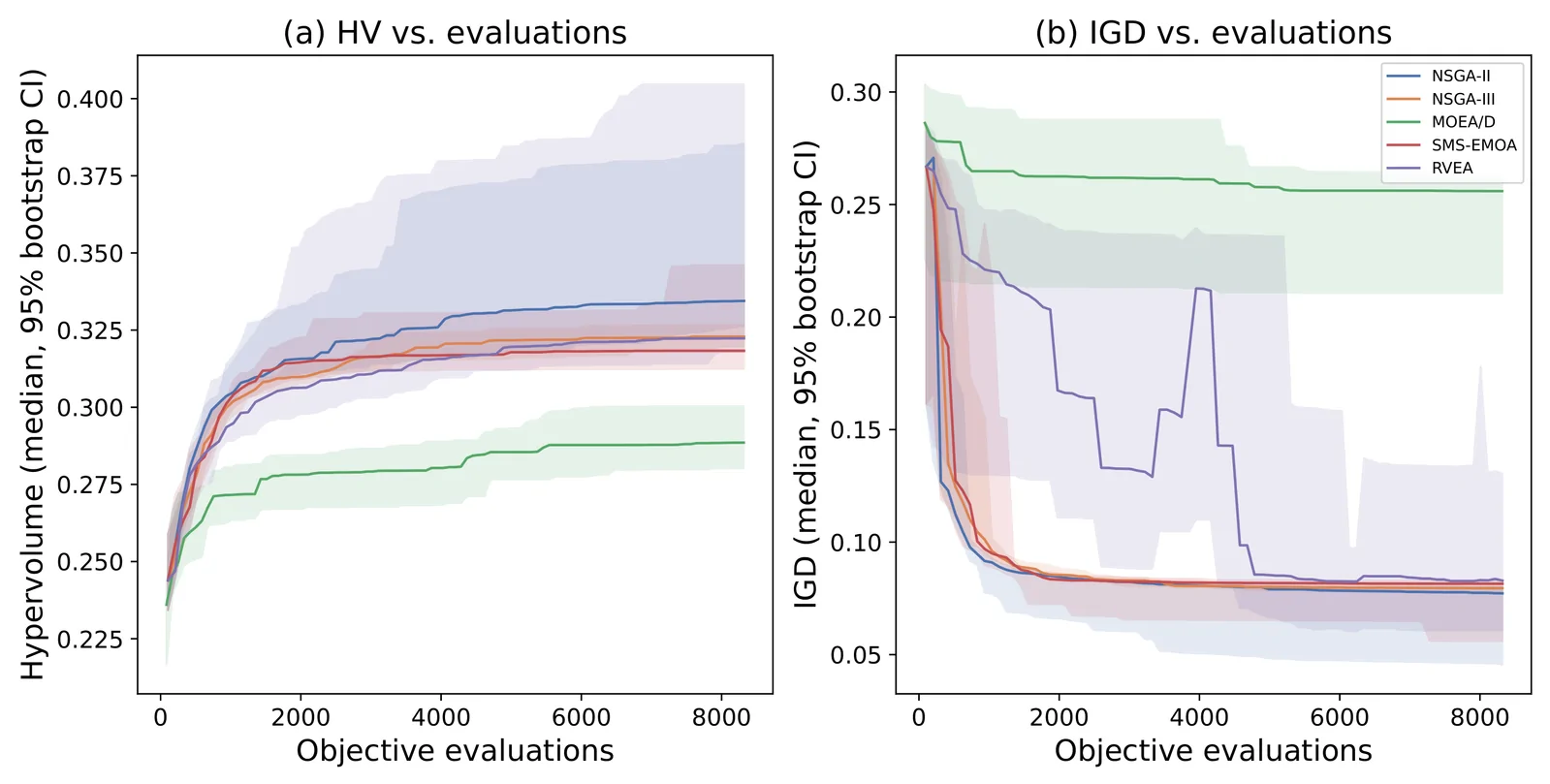
Best-so-far archive hypervolume and IGD vs. objective evaluations for all five algorithms (10 seeds, median with 95% bootstrap CI band). All five plateau well before the 8316–8320-evaluation budget ends, including MOEA/D once convergence is tracked on the cumulative archive rather than the current population’s own front.

The main comparison uses final-generation fronts only; to characterise how quickly each algorithm approaches that front, we separately track HV/IGD per generation for the five original benchmark algorithms (10 seeds, same matched budget, same normalisation and IGD reference as the main comparison—see Figure 2). Each generation’s HV/IGD is computed on a genuine best-so-far archive: the non-dominated frontier of every solution evaluated up to and including that generation (the union of the running archive and the current population’s own evaluations, re-filtered to non-dominance and deduplicated every generation), not on the current population’s own front (pymoo’s algorithm.opt or pop) in isolation. This guarantees HV is non-decreasing by construction—verified directly on the per-seed traces, exactly monotone for all five algorithms—since the non-dominated frontier of a growing candidate pool can only match or improve the previous frontier’s dominated volume. Archive-IGD is not subject to the same guarantee (removing a now-dominated point can occasionally leave a particular reference point’s nearest surviving neighbour slightly farther away, even though the archive improved in the Pareto sense). Checked directly on all 50 per-seed traces against the 561-point reference: the large majority of generation-to-generation steps either decrease or change negligibly when they increase (only 4.7% of steps increase at all; median increase $2\times 10^{-5}$, 90th percentile $9\times 10^{-4}$), but a small number of individual steps—concentrated in NSGA-II, NSGA-III, SMS-EMOA and RVEA, not MOEA/D—show a substantially larger one-generation jump (up to 0.14, against a global IGD range of roughly 0.01–0.31 across all five algorithms and all generations). We report this honestly rather than as “negligible”: archive-IGD against this front is not uniformly well-behaved generation-to-generation, even though it is non-increasing in aggregate and the algorithm ranking it supports (Section 6.1) does not depend on any single generation’s value. All five algorithms reach at least 97.2% of their own final-budget median HV by half the evaluation budget (4200–4264 of 8316–8320 evaluations): NSGA-II 98.6%, NSGA-III 99.3%, MOEA/D 97.2%, SMS-EMOA 99.6%, RVEA 98.1%. The 80/99-generation budget used throughout this paper is therefore generous rather than tight for all five algorithms under this decoder and oracle—none, including MOEA/D, is still improving substantially when the run ends once convergence is measured on a genuine best-so-far archive rather than the current population’s own (occasionally regressing) front. MOEA/D’s archive-IGD in particular decreases smoothly and monotonically throughout the run (0.286→0.261→0.256 at 1/50/100% of budget, its own largest single-generation increase only $8\times 10^{-5}$); an early sharp IGD rise followed by a persistent plateau is visible only when the current population’s own front is tracked instead of the archive, since MOEA/D’s population repeatedly reconverging onto a small handful of distinct objective vectors (Section 6.1) degrades the *population*’s own IGD even while the archive of everything it has ever found keeps improving—a good illustration of why the archive, not the live population, is the metric that answers “is the search still improving.”

### 6.2. Pareto Front Structure

**Figure 3:**
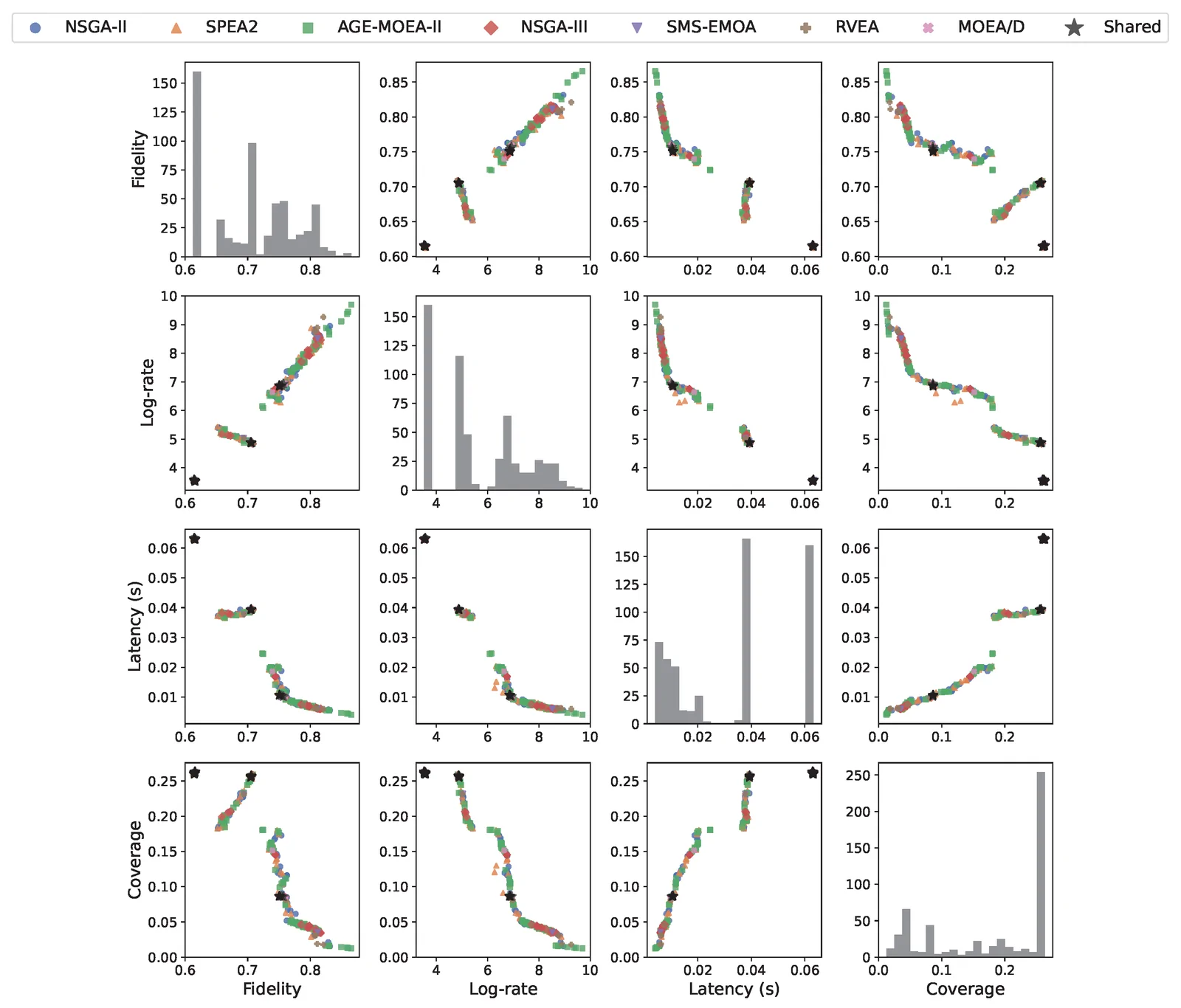
Pairwise projections of the pooled evolutionary front (union of all seven algorithms, NSGA-II, NSGA-III, MOEA/D, SMS-EMOA, RVEA, SPEA2 and AGE-MOEA-II, 30 seeds each, 561 deduplicated non-dominated points), coloured by source algorithm, with points independently (re)discovered by more than one algorithm shown as a separate “Shared” category (black stars) rather than arbitrarily attributed to a single algorithm. Rate/latency correlation ρ=−0.974, rate/coverage correlation ρ=−0.959, fidelity/rate correlation ρ=+0.972.

The pooled evolutionary non-dominated front—the union of all seven algorithms (NSGA-II, NSGA-III, MOEA/D, SMS-EMOA, RVEA, SPEA2 and AGE-MOEA-II) across all 30 seeds each, pooled and filtered to mutual non-dominance—contains 988 raw (algorithm, seed, point) records. Deduplicating by objective vector (rounded to $10^{-6}$, the natural unit for a Pareto front, which is defined in objective space) reduces this to 561 genuinely distinct points (Figure 3); the same solution is frequently rediscovered by the same or a different algorithm across seeds, and counting each rediscovery as an independent contribution would overstate diversity. Of the 561 unique points, 118 (21.0%) are found exclusively by AGE-MOEA-II, 109 (19.4%) exclusively by SMS-EMOA, 97 (17.3%) exclusively by SPEA2, 96 (17.1%) exclusively by NSGA-II, 63 (11.2%) exclusively by NSGA-III, 12 (2.1%) exclusively by RVEA, and 10 (1.8%) exclusively by MOEA/D, with the remaining 56 (10.0%) found by more than one algorithm; full per-point (algorithm, seed) provenance is released with the reproducibility package (global_pareto_v3_provenance.json). No single algorithm dominates the front’s exclusive contributions the way NSGA-II’s aggregate HV/IGD lead (Section 6.1) might suggest: SMS-EMOA and SPEA2 each contribute a share comparable to or larger than NSGA-II’s, consistent with the top-group and near-top algorithms exploring genuinely different, only partially overlapping regions of objective space rather than one algorithm’s front subsuming the others’. Separately, decoding every one of the 988 raw records reveals 944 structurally distinct topologies (distinct edge sets)—fewer than the 988 raw records (some rediscovery collapses to the same topology, as expected) but more than the 561 unique objective vectors, since occasionally two different decoded topologies map to numerically identical objective values; we therefore report the Pareto *front* (objective space, 561 points) and the Pareto *set* (design space, 944 topologies) as related but distinct quantities, rather than treating either count as uniquely “the” size of the solution set. The four objectives span physically meaningful ranges: fidelity $\bar{F}\in \left[0.612,0.866\right]$, log-rate ${\bar{R}}_{\log}\in \left[3.52,9.70\right]$, latency $\bar{L}\in \left[0.004,0.063\right]$ s and coverage $C_{\tau }\in \left[0.013,0.263\right]$. Table 8 additionally reports edge count and medium composition across this front, since a wider or denser topology could in principle achieve better objective values simply by using more infrastructure than the classical baselines it is compared against in Section 6.4.

#### How Connected Are These Topologies?

Because $\bar{F}$, ${\bar{R}}_{\log}$ and $\bar{L}$ are averaged only over *connected* city pairs (Section 3), a topology that simply fails to connect its hardest pairs removes them from three of the four objectives and pays only in coverage. This is a real property of the formulation, and it has a measurable consequence for how the released front should be read, so we report the connectivity structure explicitly rather than leaving it implicit in the edge counts. (connectivity_diagnostics_v3.py; connectivity_diagnostics_v3.json. Connectivity is computed on the same best-medium adjacency the oracle routes over.) Of the 561 front points, **none** connect all 100 cities in a single component under the satellite-visibility-corrected oracle (Section 4.2); the median point has five connected components and a giant component of 66 cities, and reaches 50.9% of the 4950 city pairs (mean 48.2%). This is a real change from an earlier, uncorrected version of the oracle, in which a single long-range satellite link could bridge otherwise-disconnected continents at no cost in the objectives that ignore unreachable pairs; capping satellite range at the physical visibility limit removes exactly that shortcut, and no topology in the released front’s search compensates for it with enough additional fibre or FSO hops to close every last component. This reflects a specific and deliberately conservative satellite configuration: a single LEO satellite per ground-to-ground hop, visible to both endpoints simultaneously (Equation (A6)), with no inter-satellite link (ISL) relay and no multi-satellite constellation modelled. A constellation with cross-links could in principle bridge two ground stations that share no common satellite pass, closing some of the disconnected components reported here; modelling that explicitly would add satellite-to-satellite hops and constellation geometry to the oracle, which we leave as future work (Section 8). Reachability is no longer bimodal in the way it was before the correction: 28.5% of points reach at least 90% of pairs, 54.0% reach at least half, and only 21.2% reach fewer than 10% (down from 28.1% under the uncorrected oracle). The degenerate, near-fully disconnected extreme also disappears entirely: no front point now has fewer than 20 stored edges, against three such points before the correction. Coverage at the fidelity threshold remains bounded well below one everywhere on the front: the best point covers 26.3% of city pairs, the median 21.2%. Edge count and reachability are almost perfectly rank-correlated (Spearman ρ=0.944, $p<10^{-270}$), so the |E| ranges in Table 8 can be read as a proxy for operational reach.

We therefore qualify the phrase “global quantum internet topology” where it is applied to the front as a whole, and more strongly than an earlier version of this analysis did: with a single satellite’s physical visibility limit correctly enforced, this benchmark’s 11-variable decoder does not find any topology that is simultaneously Pareto-optimal on fidelity/rate/latency/coverage *and* fully connects all 100 cities. Most points reach a majority of city pairs, and a quarter reach nearly all of them, but closing the last components appears to trade against the other objectives sharply enough that the evolutionary search does not retain such solutions on the front. A design study that requires full connectivity should filter the released front by component count, or add it as an explicit constraint to the optimisation itself rather than reading it off the unconstrained front; we have not attempted the latter here. Whether a multi-satellite constellation—which we do not model (Appendix A.2)—would restore fully connected solutions to the Pareto-optimal set is a natural question this single-satellite benchmark cannot answer.

The pairwise correlations show two different relationships, not one uniform trade-off. Rate and latency are *both* minimised-cost or maximised-benefit in a way that moves together here: since latency is minimised and rate is maximised, their strong negative correlation (ρ=−0.974) means higher-rate topologies tend to *also* have lower (better) latency, not worse—the two are substantially aligned on this front rather than in tension, likely because both benefit from the same short, high-fidelity links (Table 8). Coverage is the objective genuinely in tension with the other three: its strong negative correlation with rate (ρ=−0.959) is a real trade-off, since both are maximised. One mechanism consistent with this, which we state as a hypothesis rather than as something these correlations establish, is a change in the averaging set: adding edges cannot lengthen the best route between a pair that was *already* connected, but it does admit new, harder pairs into P, and those pairs enter the means with long routes and low rates. The connectivity diagnostics above are consistent with this reading—reachability and edge count are almost perfectly rank-correlated—but distinguishing it from alternatives would require per-pair route-length and composition analysis across the front, which we have not performed. Fidelity and rate are strongly *positively* correlated on this front (ρ=+0.972): topologies that achieve high per-pair fidelity under the canonical oracle’s harsher satellite/FSO fidelity constants (Section 4.2) tend to also achieve higher rates rather than trading one against the other. A plausible common cause is edge composition (Table 8: predominantly satellite by pooled edge count, 87.1%, but with a substantially larger fibre share than a naive distance-only latency model would suggest), again untested here as a mechanism; the descriptive statement—that the evolutionary search converges on this composition across all seven algorithms—is what the data support.

### 6.3. Categorical Edge-And-Medium Baseline

A central design choice of this work is the 11-variable mixed parametric decoder rather than direct optimisation over the 4950-edge candidate set. To empirically substantiate this choice, we ran NSGA-II and NSGA-III with an identical evolutionary budget (population 104, 80 generations, SBX crossover, polynomial mutation, biased sparse initialisation ∼3% active edges) directly on the edge set, using the same oracle and four objectives. A binary or medium-fixed edge-mask baseline is representationally unfair to compare against the parametric decoder if every active edge is forced onto a single fixed medium (e.g., always fibre, or always the argmax-fidelity medium for that city pair): the decoder can select a lower-fidelity medium when it trades off better on rate, latency or coverage, but a fixed-medium rule cannot. Here, each of the 4950 city pairs is instead a genuinely free categorical variable in {no edge, fibre, satellite, FSO}, searched via a real-valued relaxation (each variable in [−0.499,3.499], rounded to the nearest integer category at evaluation time) under the same SBX/PM operators used elsewhere in this paper—a standard pragmatic encoding for categorical variables under continuous operators, documented here rather than hidden. This 4950-variable search is markedly *faster* per run than the 11-variable decoder (0.63–0.71 vs. 4.56–4.87 min, Table 9—fewer feasible satellite edges for the unguided per-pair search to consider once satellite range is capped), not comparable in the ordinary sense; 30 seeds are used here for statistical-power parity with the main comparison, not because the two run at similar cost. HV, IGD and spacing are computed against the same combined, deduplicated reference front used for both algorithms (not only $|F^{★}|$, which alone does not indicate front quality). We caution that this demonstrates a naive categorical relaxation under SBX/polynomial mutation performs poorly at this evaluation budget; the paragraph below tests category-native operators (uniform crossover, add/remove/change-medium mutation) directly and finds they narrow but do not close the gap, so a linkage-learning approach or a different budget regime remains untested and could plausibly do better still.

Table 9 reports the result.

Allowing the edge-and-medium baseline to select any of the four candidate states (no edge, fibre, satellite, FSO) per city pair—rather than a single fixed medium—does not close the gap with the parametric decoder—if anything, the gap remains substantial once both representations are scored against the same, deduplicated reference front. HV drops by roughly a factor of 5 (0.64–0.66 for the decoder vs. 0.124–0.125 for the categorical baseline) and IGD rises from 0.04–0.05 to over 0.66. This factor is markedly smaller than the ∼58× an earlier, uncorrected version of the satellite model reported for the same comparison. A plausible explanation, consistent with but not established by this result, is that the categorical baseline’s naive per-pair search benefited disproportionately little from that model’s unrealistically long-range satellite links, which the *k*-nearest-neighbour decoder could reach more reliably by construction, inflating the decoder’s advantage; capping satellite range at the physical visibility limit (Section 4.2) would then have removed that unfair advantage. We have not isolated this from the other three changes made to the link model at the same time (two-arm efficiency, latency, the removed rate floor), so we report it as a hypothesis rather than a demonstrated cause; the robust fact is that a real, 5× gap remains under the corrected oracle. Unique-solution cardinality tells a more mixed story than before: the categorical baseline under NSGA-II (64.6±16.5) is now statistically comparable in scale to the decoder’s own cardinality (89.9), though still markedly more variable across seeds and with a substantially wider IGD, while NSGA-III’s categorical cardinality (10.1±2.1) remains an order of magnitude below the decoder’s (65.1). The categorical search’s wall-clock time per run also fell sharply under the corrected oracle (0.6–0.7 min, against 4.7–4.9 for the decoder), consistent with far fewer feasible satellite edges for the unguided per-pair search to consider once satellite range is capped. We still read the surviving 5× HV gap and the much worse IGD as evidence that the curse of dimensionality in a 4950-variable search space, not medium-assignment fairness, remains a real bottleneck: SBX/PM operators derived for low-dimensional continuous spaces do not scale as well to a 4950-variable relaxed-categorical encoding under this evolutionary budget as they do to the 11-variable decoder, regardless of how freely each variable can express medium choice—but the earlier, much larger factor overstated how dominant that bottleneck is relative to the physical model’s own influence on the comparison.

#### 6.3.1. A Second Categorical Baseline with Category-Native Operators

The comparison above uses a real-valued relaxation of the categorical representation under continuous operators (SBX/polynomial mutation); we additionally test operators native to the discrete representation itself—uniform categorical crossover and add-edge/remove-edge/ change-medium mutation, no relaxation or rounding—with NSGA-II at four initial edge densities (3%, 10%, 10.9%, and 15%); the 10.9% setting was chosen to sit near the decoder’s own pooled-front median density as measured before the satellite-visibility correction, and no longer does (19.6% under the corrected oracle, median 971 of 4950 possible city-pair edges, Table 8)—we report the original four densities as run rather than adding a fifth after the fact, since 3% through 15% already spans the relevant range; 10 seeds each, matched budget). Within this second baseline, initial density matters enormously, but in the *opposite* direction from an earlier, uncorrected version of this oracle: mean HV (own 4-density-pooled reference, 30 seeds at the best density) is highest at 3% (0.252±0.030) and falls sharply with density (0.017±0.004 at 10%, 0.019±0.004 at 10.9%, 0.028±0.003 at 15%), with IGD lowest at 3% (0.296) and above 0.95 at every denser setting. A denser initial population now carries more now-infeasible long-range satellite candidates for the add-edge/remove-edge/change-medium mutation to discover and discard, which we read as the likely mechanism, though we have not isolated it directly. Scored against a reference front shared with the decoder (this comparison’s own 10 seeds, not Table 9’s 30), the best-performing density (3%) reaches HV =0.148±0.019 versus the decoder’s 0.635±0.052 under identical seeds and budget—a ∼4.3× gap, versus ∼5× for the relaxed SBX/PM baseline (Table 9): category-native operators at their best density now close slightly *more* of the gap to the decoder than the real-valued relaxation does, a reversal from an earlier, uncorrected version of this comparison in which SBX/PM came markedly closer. Category-native operators at a well-chosen density substantially narrow the gap but do not close it: the compact parametric decoder still dominates direct categorical optimisation at this 4950-variable scale under both operator families and under whichever density each family does best at.

#### 6.3.2. A Single Master Comparison, One Shared Reference Front

The two categorical baselines above (and Table 9) each use their own block-specific reference front and normalisation, following this paper’s general convention (Section 5)—correct for within-block comparisons, but not directly comparable across blocks without care. As a single, unambiguous cross-check, we additionally pool the decoder, the relaxed-categorical SBX/PM baseline, and the best-performing category-native-operator density (3%, now also raised to 30 seeds for parity with the other two rows) into ONE shared, deduplicated reference front and normalisation (Script: representation_comparison_master_v3.py). Under this single scale: decoder HV=0.696±0.044, category-native (d03) HV=0.157±0.020 (∼4.4× gap), relaxed-categorical SBX/PM HV=0.132±0.022 (∼5.3× gap). A Kruskal–Wallis omnibus test across the three is significant (H=66.200, $p<10^{-14}$), and all three pairwise Mann–Whitney comparisons are significant ($p<10^{-4}$ throughout), including category-native vs. relaxed-categorical ($p=9.2\times 10^{-5}$): under this shared scale, the category-native operators at their best density now significantly *outperform* the SBX/PM relaxation, reversing their relative order under the previous, uncorrected oracle. The qualitative headline conclusion is unchanged from the block-specific numbers above—the decoder dominates both categorical alternatives—but which categorical alternative comes closer to it, and by how much, both changed once the satellite geometry was corrected: the best category-native density flipped from 15% to 3% (Section 6.3 above explains why), and category-native operators no longer merely narrow the gap to relaxed-categorical SBX/PM—at their best density, they close it and go slightly past. The exact gap magnitude reported here should be read as the authoritative one where it differs from the block-specific numbers, since only this comparison holds the reference front and normalisation fixed across all three representations simultaneously.

### 6.4. Classical Network-Design Baselines

**Figure 4:**
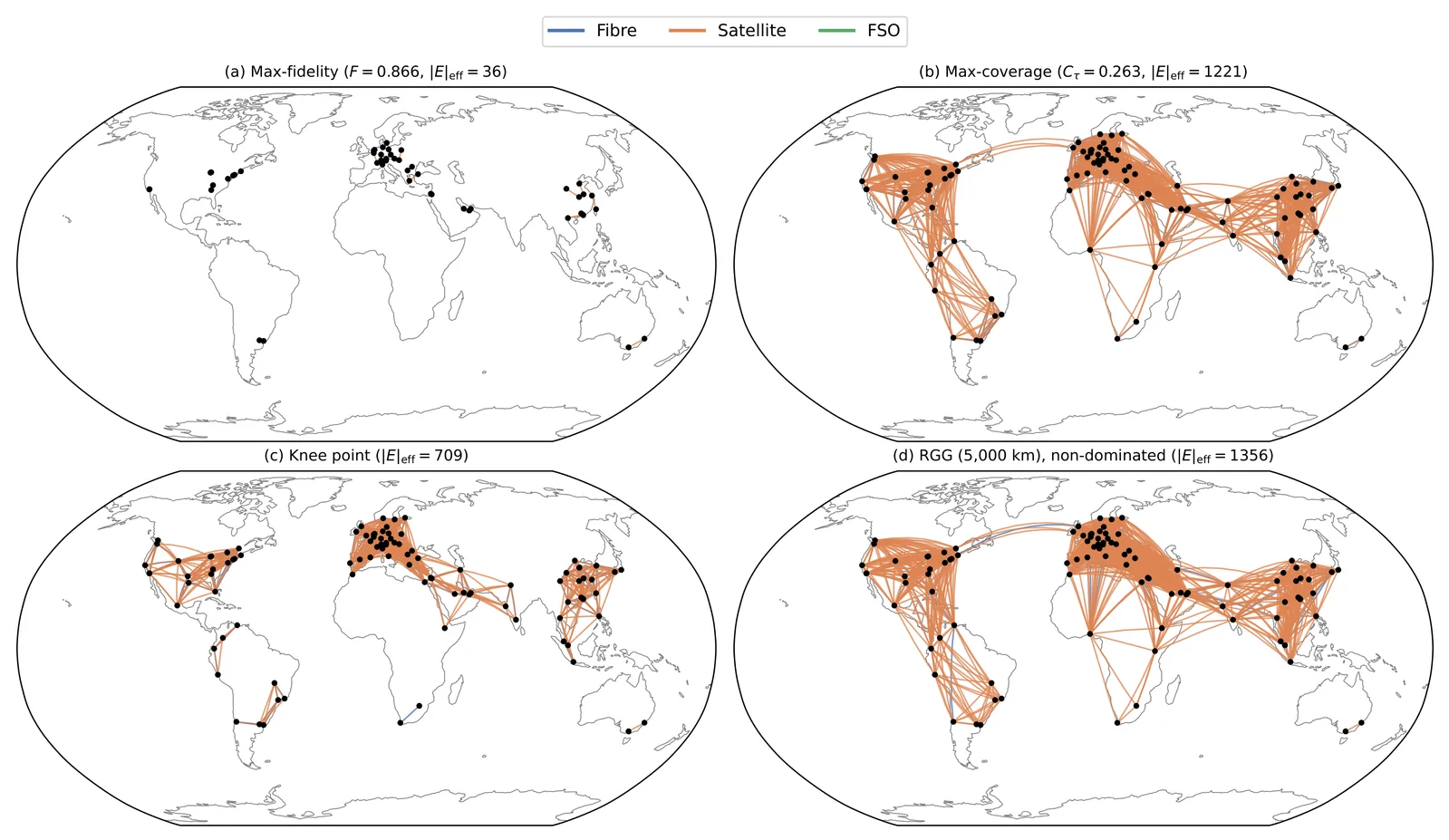
Four representative topologies, coloured by medium. No FSO (green) edge appears in any of the four panels: FSO is available to the decoder but is selected only marginally, consistent with the ablation of Section 6.5; the entry is retained in the legend because the same colour key is used for every topology figure in this paper. |E| is reported as the effective, oracle-routed count throughout (unique city pairs actually connected; Table 8), the only value directly comparable across all four panels. (**a**) The pooled front’s maximum-fidelity solution (F=0.866, ${\left|E\right|}_{\mathrm{eff}}=36$): sparse, regional, fidelity-optimised at the cost of coverage. (**b**) The maximum-coverage solution ($C_{\tau }=0.263$, ${\left|E\right|}_{\mathrm{eff}}=1221$): dense, near-global, predominantly satellite. (**c**) The knee point (closest to the ideal corner in normalised objective space, ${\left|E\right|}_{\mathrm{eff}}=709$): a denser intermediate design, reaching markedly higher coverage than (**a**) at a real cost in fidelity. (**d**) The non-dominated classical constructor—a Random Geometric Graph at a 5000 km threshold (${\left|E\right|}_{\mathrm{eff}}=1356$, Table 10)—a fundamentally different, threshold-based structure the parametric decoder’s *k*-nearest-neighbour rules do not generate, reaching higher coverage than any evolutionary solution on the front. (Under an earlier, uncorrected version of the satellite model, the non-dominated classical exemplar shown here was a 10-hub hub-and-spoke network; Section 6.4 explains why that changed.) Selection indices and exact objective values are released with the reproducibility package.

To test whether the evolutionary framework produces topologies superior to deterministic heuristics from classical network design, we evaluated 14 classical topology constructors with the same canonical oracle: a global Minimum Spanning Tree (MST) over geodesic distances; *k*-nearest neighbours for k∈{3,5,7,10}; Random Geometric Graphs with distance thresholds in {500,1000,2000,3000,5000} km; hub-and-spoke topologies with {5,10,15} hubs selected by MST degree centrality; and a region-aware MST (per-region MST plus inter-region bridges). Each edge is assigned its argmax-fidelity medium (fibre, satellite or FSO), so every classical constructor can draw on all three media rather than being restricted to fibre. This is a substantial fairness improvement over a fibre-only baseline, but it is not fully equivalent to the decoder’s freedom: argmax-fidelity is a fixed, greedy per-edge rule, whereas the decoder can select a lower-fidelity medium when it trades off better on rate, latency or coverage, which a fixed rule cannot. Figure 4 makes the structural difference visible, placing three representative solutions from the pooled evolutionary front alongside the non-dominated classical constructor.

Table 10 reports all 14 constructors across the five families, re-checked for Pareto dominance directly against the 561-point deduplicated pooled evolutionary front of Section 6.2. Of the 14 classical constructors, 13 are dominated by the pooled evolutionary front; the remaining one—the Random Geometric Graph at a 5000 km threshold, marked †—is itself non-dominated, meaning no evolutionary solution simultaneously matches or beats it on all four objectives. This is a substantial change from an earlier, uncorrected version of this oracle, in which all three hub-and-spoke variants were the non-dominated classical constructors instead: hub-and-spoke’s coverage advantage depended on a small set of hub nodes reaching every other city directly, predominantly over long-range satellite links, and capping satellite range at the physical visibility limit (Section 4.2) removes most of those links outright. The 15-hub baseline’s coverage collapses from $C_{\tau }=0.559$ under the uncorrected oracle to 0.177 here, well inside the evolutionary front’s own range, and all three hub-and-spoke variants are now dominated. The RGG family is less exposed to this because its edges are threshold-based rather than hub-concentrated, and at 5000 km, it still reaches $C_{\tau }=0.278$—narrowly above the evolutionary front’s own observed maximum coverage (0.263, Table 10 last row)—which is sufficient on its own for no front point to dominate it, since dominance requires matching or beating every objective simultaneously. Being dominated on these four objectives does not make hub-and-spoke uncompetitive in practice: a hierarchical, core/access design with *h* hubs needs on the order of h(N−h) access links plus a small core mesh among the hubs, against $O(N^{2})$ candidate links for an unconstrained topology, so its construction and maintenance cost—physical fibre runs, ground-station sites, or licensed satellite passes, none of which this oracle prices—can be substantially lower even where its fidelity, rate, latency and coverage are all worse. None of the four objectives evaluated here is a cost proxy, so the dominance result above should be read as “worse on the physical performance axes we measure,” not as “economically worse”: a full comparison would need the deployment-grade infrastructure-cost objective already flagged as future work (Section 8), which could plausibly return some hub-and-spoke variants to the non-dominated set once cost is scored alongside performance. We read the decoder as exposing a broad but demonstrably non-exhaustive subset of the feasible trade-off surface, not the full Pareto-optimal set: it strongly outperforms the evaluated alternative representations and operators everywhere it reaches, but the single highest-coverage region of the feasible surface currently sits just outside that reach and would require either a search bias toward denser, more RGG-like topologies or an explicit coverage-floor constraint to access.

### 6.5. Decoder Ablation

Every variant below is run with a single algorithm, NSGA-III, rather than repeating the full seven-algorithm comparison eight times over: at 30 seeds per variant, this keeps the ablation’s total evaluation budget in the same order of magnitude as the main comparison instead of multiplying it eightfold. NSGA-III was chosen over the top-ranked NSGA-II (Section 6.1) specifically because it is the more conservative choice for a medium-necessity claim: NSGA-III is significantly separated *below* the top group on the main comparison, so a medium shown necessary here is necessary for an algorithm that is not already getting the most out of the representation, making the finding harder to dismiss as an artefact of pairing a weak ablation baseline with an already-strong optimiser. As stated as a limitation in Section 8, this choice means the medium-necessity ordering established below is confirmed only for NSGA-III and has not been re-tested on NSGA-II, SPEA2 or AGE-MOEA-II; we picked one principled, disclosed baseline over an unprincipled eightfold budget increase, rather than leave the choice unstated.

**Figure 5:**
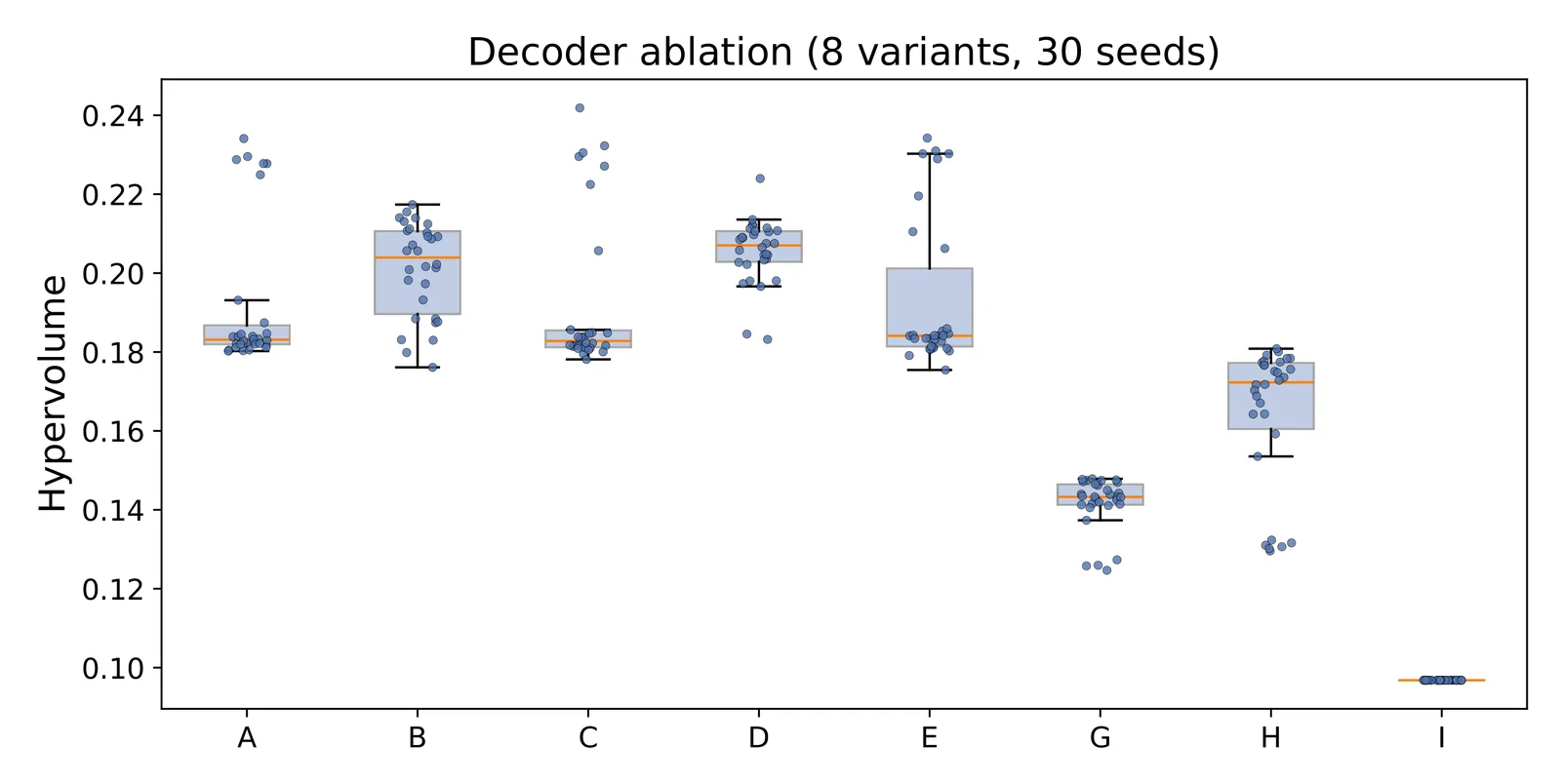
Decoder ablation (eight variants, 30 seeds, media hard-disabled rather than parameter-constrained—see Section 6.5). Boxes show median/IQR with jittered per-seed points overlaid, as in Figure 1. Variants lacking fibre (G, satellite-only; H, satellite+FSO; I, FSO-only) all show significantly lower hypervolume than the full decoder ($p_{\mathrm{Holm}}\leq 10^{-5}$ throughout); FSO-only (I) collapses most severely of all. Fibre-only (B), fibre+satellite (C) and no-inter-region-bonus (E) are statistically indistinguishable from the full decoder (A); fibre+FSO (D) is significantly *higher* than the full decoder.

We evaluate eight variants of the decoder (Table 11, Figure 5): (A) the full decoder; (B) fibre-only ($k_{s}=k_{g}=0$); (C) fibre+satellite ($k_{g}=0$); (D) fibre+FSO ($k_{s}=0$); (E) no inter-region bonus ($\beta_{r}=0$); (G) satellite-only ($k_{f}=k_{g}=0$); (H) satellite+FSO ($k_{f}=0$); (I) FSO-only ($k_{f}=k_{s}=0$). Variants G, H and I complete the single-medium and dual-medium sweep (every one- and two-medium combination is now tested), which lets the ablation distinguish “a given medium is necessary alongside the others” from “that medium is sufficient alone.” Each variant runs NSGA-III with 30 seeds, and hard-disables every non-enabled medium’s entire edge-generation step (rather than only constraining its decoder parameters), so a disabled medium contributes exactly zero edges to the decoded topology.

#### 6.5.1. Interpretation

A Friedman omnibus test across all eight variants’ HV values, paired by seed, is highly significant ($\chi^{2}=167.289$, $p<10^{-32}$), justifying the Holm-corrected paired Wilcoxon comparisons against variant A that follow. With media genuinely hard-disabled (rather than merely parameter-constrained—see Section 6.5) and 30 paired seeds, the ablation supports a clear, specific conclusion, unchanged by the satellite-geometry correction of Section 4.2: *under NSGA-III, fibre is necessary, but satellite’s contribution beyond fibre alone is not statistically detectable at this benchmark, oracle and search-range configuration*. Removing fibre (G, satellite-only; H, satellite+FSO; I, FSO-only) significantly collapses HV in every case ($p_{\mathrm{Holm}}<1.4\times 10^{-8}$ throughout; Cliff’s δ=+1.00, +0.99 and +1.00 respectively, matched-pairs rank-biserial $r_{rb}=+1.00$, +1.00 and +1.00): no variant lacking fibre matches the full decoder. By contrast, removing satellite (B, fibre-only) does *not* significantly change HV relative to the full decoder ($p_{\mathrm{Holm}}=0.088$, δ=−0.44, $r_{rb}=-0.45$)—if anything, the point estimate is slightly higher for fibre-only (HV=0.201) than the full decoder (0.192), though not significantly so. Fibre+satellite (C, HV=0.193) is likewise statistically indistinguishable from the full decoder ($p_{\mathrm{Holm}}=1.000$, δ=+0.07). Unexpectedly, fibre+FSO (D, satellite hard-disabled, HV=0.206) is significantly *higher* than the full decoder ($p_{\mathrm{Holm}}=0.003$, δ=−0.57, $r_{rb}=-0.67$). A plausible but untested hypothesis is that removing satellite (which the decoder’s search bounds allow far more candidate edges for than fibre or FSO, Table 2) simplifies the search landscape enough, under a fixed 80-generation budget, to more than offset satellite’s own contribution to fidelity/rate; this is not established here and would need a dedicated follow-up. FSO-only (I) collapses most severely of all variants (HV=0.097, $p_{\mathrm{Holm}}<1.4\times 10^{-8}$, δ=+1.00), consistent with the geometry of the benchmark: only 11 of the 4950 candidate city pairs (0.2%) fall within FSO’s 150 km range given the 100-city global distribution used here, so we read FSO-only’s collapse as reflecting this specific sparse global-city benchmark and short-range FSO model, not a general claim that FSO is unimportant for quantum networks at every scale—a metropolitan-density benchmark with many short-range city pairs could plausibly show a different result. The inter-region bonus variant (E) is statistically indistinguishable from the full decoder ($p_{\mathrm{Holm}}=1.000$, δ near zero), with essentially identical mean HV (0.1935 vs. 0.1922), so we do not claim the inter-region bonus is harmful or helpful. We caution that the ablation block uses its own block-specific normalisation, so its absolute HV values are not comparable to the algorithm-comparison block’s HV (Section 5).

#### 6.5.2. The Symmetric IGD_union_ Ranks Variants Differently from HV

Because the primary IGD column is measured against variant A’s own front, it trivially favours A; the symmetric IGD_union_ (Table 11, against the shared union of all eight variants’ fronts, now computed per-seed over the same 30 seeds like every other metric) tells a partly different story. Satellite+FSO (H, ${\mathrm{IGD}}_{\mathrm{union}}=0.1855\pm 0.0636$) is closer to the shared union front than the full decoder itself (0.2282±0.0486) or fibre+satellite (0.2270±0.0493), even though H’s HV (0.1644) is well below A’s and C’s. This is not a contradiction: HV measures dominated volume against a fixed reference point and rewards extent and spread, whereas IGD_union_ measures average proximity to the pooled front and can favour a variant whose narrower front happens to sit close to a densely populated region of the union (here, the satellite/FSO-dominated corner contributed disproportionately by variants B, D, G, H and I) without covering as much of the objective space overall. We report this as a genuine, if secondary, structural observation about the two indicators rather than revising the HV-based conclusions above.

### 6.6. Sigma Ablation

The decoder’s eleventh variable, σ (seed offset), is a pseudorandom-number-generator seed rather than a physical design parameter (Threats to validity, item vii): it selects which realisation of the decoder’s stochastic edge-sampling process is decoded, so two numerically close σ values need not produce similar topologies. We test directly whether the search benefits from treating σ as a free, optimised variable by comparing five NSGA-III variants at matched budget (population 104, 80 generations, 15 seeds 42–56): (A) the standard 11-variable decoder used throughout this paper, with σ optimised; (B) a genuinely 10-variable decoder (σ is never a decision variable the search algorithm’s own crossover/mutation operators see) with σ hard-fixed to one of three constants tested independently (0, 42, 500), rather than a single arbitrary choice; and (C) a genuinely 10-variable decoder whose fitness is the mean objective vector over K=3 independent realisations (σ∈{0,1,2}) of the same physical parameters, rather than a single realisation. Table 12 reports the result.

A Kruskal–Wallis omnibus test across all five variants’ HV values is significant (H=37.943, $p=1.2\times 10^{-7}$). Under Holm-corrected pairwise Mann–Whitney tests (independent samples, 10 comparisons), A is statistically indistinguishable on HV from the two better-performing fixed values ($p_{\mathrm{Holm}}=1.000$ against both σ=42 and σ=500) but significantly *better* than the worse one ($p_{\mathrm{Holm}}=0.015$ against σ=0, Cliff’s δ=+0.66): optimising σ matches the fixed values that happen to be good and clearly beats the one that happens to be bad. Critically, the fixed values are not interchangeable with *each other*: σ=0 is significantly worse than both σ=42 ($p_{\mathrm{Holm}}=0.035$, δ=−0.58) and σ=500 ($p_{\mathrm{Holm}}=0.033$, δ=−0.60), while σ=42 and σ=500 are themselves indistinguishable ($p_{\mathrm{Holm}}=1.000$). This is the central finding of testing multiple fixed values rather than one: fixing σ removes a free search dimension, but the resulting performance is sensitive to *which* constant is chosen, and nothing in the decoder’s own structure indicates in advance which constant will be favourable (there is no a priori reason to expect σ=500 to outperform σ=0; both are arbitrary points in the same 1000-value domain). Optimising σ as a free variable (A) sidesteps this guessing problem entirely: it lands statistically level with whichever fixed constants turn out to be favourable, without needing to know in advance which those are, and without the downside risk of landing on an unfavourable one. We still do not overstate what this shows: A’s mean HV (0.447±0.051) sits between the two good fixed values (σ=500, 0.451±0.053; σ=42, 0.439±0.039) and is not shown to *exceed* either of them—the claim is parity with the good outcomes and protection from the bad one, not a performance advantage over a correctly chosen constant. Averaging over K=3 realisations (C) remains significantly worse than every other variant, including the worst individual fixed constant ($p_{\mathrm{Holm}}\leq 0.046$ throughout, $p_{\mathrm{Holm}}<10^{-4}$ against A and the two good fixed values), consistent with an averaged fitness diluting the gradient the search would otherwise get from occasionally sampling a favourable realisation, at 3–4× the wall-clock cost of every other variant (11.7 vs. 2.9–3.5 min per run) since averaging pays for K=3 oracle calls per individual evaluated. We do not remove σ from the decoder used throughout the rest of this paper, since doing so would require re-running every other result for consistency and—given the fixed-value sensitivity just demonstrated—would require first establishing which constant to fix it to, which this ablation does not resolve; we instead report this as a validated methodological finding for future work building on this decoder.

### 6.7. Multi-Instance Validation

Every result so far uses the same 100-city benchmark instance. The seed budgets differ by experiment: 30 seeds for the algorithm comparison and the decoder ablation, 15 for the sigma ablation, and 10 for the convergence tracking and for all four instances below, including the N=200 instance (extended from an initial 5 seeds for parity with the other three). Runs on one instance do not by themselves establish that the algorithm ranking generalises to other city sets. We test this on four additional instances (three subsets and one scale-up, counted consistently as four throughout this paper). Three are real-city *subsets*, distinct from the main benchmark by size and/or geographic concentration (genuine subsets of the same verified 100-city list—no coordinates invented): *global_n50* (50 cities, every other city of the main list, preserving global spread at half the size); *regional_eu_na* (50 cities, Europe and North America only, two regions instead of five); and *metro_europe* (30 cities, Europe only, the most geographically concentrated subset available). Each runs all five algorithms with 10 seeds at the same matched budget as the main comparison (population 104, 80/99 generations). The fourth, *global_n200*, is a genuinely independent scale-up rather than a subset: 100 additional real cities sourced from the GeoNames cities15000 export [71], distributed under CC BY 4.0 and used here with attribution (real coordinates, verified against the official download and cross-checked against Wikipedia for a sample including two disambiguation-risk cases; full sourcing methodology, download date and per-city provenance accompany the reproducibility package (cities.csv; the columns derived from GeoNames are marked as such and separated from this paper’s own region assignments)), appended to the original 100 to reach N=200—correctness verified directly by confirming the first 100 rows’ pairwise distances and per-medium link tables are numerically identical to the original 100-city oracle before trusting any result.

The broader qualitative ranking is only partly stable across the four additional instances, and less stable than under an earlier, uncorrected version of the satellite model. MOEA/D is last by mean HV on all four instances, the one pattern that holds throughout. NSGA-II and NSGA-III are the top two algorithms on three of the four instances (*regional_eu_na*, *metro_europe*, *global_n200*), but not on *global_n50*, where RVEA’s mean HV (0.318) exceeds NSGA-III’s (0.309), displacing it into third place behind NSGA-II (0.331); SMS-EMOA is fourth and MOEA/D last there as elsewhere. Neither “SMS-EMOA is always third” nor “RVEA is always fourth” holds under the corrected oracle: SMS-EMOA places third on *regional_eu_na* and *global_n200* but fourth on *global_n50* and *metro_europe*, and RVEA’s rank ranges from second (*global_n50*) to fourth (*regional_eu_na*, *global_n200*). We report this instance-dependence directly rather than as a footnote: the qualitative ranking below NSGA-II/NSGA-III/MOEA/D that a five-instance uncorrected oracle had suggested was stable does not survive correcting the satellite model, and a reader should not extrapolate SMS-EMOA’s or RVEA’s rank from one instance to another on this benchmark family. On *global_n200* specifically, NSGA-III’s mean HV (0.451) now exceeds NSGA-II’s (0.433) under the all-five-algorithm reference front, the same reversal already seen on *metro_europe* (NSGA-III 0.049 vs. NSGA-II 0.037) and no longer confined to the smallest instance.

The four per-instance NSGA-II-vs.-NSGA-III comparisons form one family of hypothesis tests and are corrected jointly (Holm), since testing the same ordering on four instances and reporting only the uncorrected *p*-values would overstate the evidence. Using each instance’s own pairwise (NSGA-II-vs.-NSGA-III-only) reference front and normalisation (multi_instance_pairwise_stats_v3.json), NSGA-II’s mean HV is higher on *global_n50* (0.373 vs. 0.347; p=0.089, $p_{\mathrm{Holm}}=0.267$; Cliff’s δ=+0.46; CI_95_ on the difference [−0.001,+0.055]), but this does *not* survive the family correction, and the CI includes zero. On *regional_eu_na*, the comparison is significant both before and after correction (0.799 vs. 0.788, p=0.0022, $p_{\mathrm{Holm}}=0.0088$, δ=+0.82; CI_95_ [−0.012,+0.035], which still straddles zero despite the significant rank test—consistent with a real but modest mean difference relative to the per-seed spread), the *only* instance that survives correction. On *metro_europe* the two are statistically indistinguishable (0.036 vs. 0.047, p=0.850, $p_{\mathrm{Holm}}=1.000$) with the point estimate reversed in NSGA-III’s favour (δ=−0.06, a small effect). On *global_n200*, now with the full 10 seeds, the same reversal appears and is, if anything, slightly more pronounced (0.439 vs. 0.454, p=0.791, $p_{\mathrm{Holm}}=1.000$, δ=−0.08); neither instance’s reversal is statistically significant, but two of four instances now point the same (NSGA-III-favouring) direction rather than one. Taken together, of the four additional instances, only one provides corrected evidence for the NSGA-II/NSGA-III ordering seen on the main benchmark, and the reversal first observed on the smallest, most geographically concentrated instance (*metro_europe*, 30 cities, all within Europe) is echoed, at comparable effect size, on the largest instance tested (*global_n200*, 200 cities, global). That the two instances at opposite ends of this benchmark’s size range share a point estimate not seen in the two mid-sized instances is worth noting but should not be over-read from only four instances and no significant effect in either direction; we do not have a mechanistic account of it. NSGA-II’s advantage on the main 100-city benchmark is driven by a minority of seeds reaching a qualitatively better basin (Section 6.1), and a plausible but untested hypothesis is that this basin-finding advantage depends on properties of the specific 100-city instance that do not transfer uniformly to other instance sizes or geographic distributions—but we did not test this specific hypothesis directly.

## 7. Discussion

### 7.1. What Transfers Beyond Quantum Networks

The application domain here is quantum-network design, but the findings that generalise are about evolutionary optimisation over graphs, and we state them as such. Representation dominates optimiser choice by a wide margin, though not the wide margin an earlier version of this analysis reported. On the same problem, the same oracle and the same budget, moving from a 4950-variable categorical encoding to an 11-variable parametric decoder changes hypervolume by a factor of roughly 4.4 against the best-performing categorical baseline (category-native operators at their best tested density) and 5.3 against the weaker relaxed-categorical SBX/PM baseline, whereas the spread across seven mature algorithms on the decoder is under 1.5× (1.43), and much of that is not statistically separable. The representation-vs-optimiser gap shrank by an order of magnitude once the satellite-visibility correction (Section 4.2) was applied—an earlier, uncorrected run of this same comparison reported a factor of roughly 58. This shrinkage is consistent with—though it does not prove—the categorical baseline’s naive search benefiting disproportionately little from the uncorrected model’s unrealistically long-range satellite links, which the *k*-nearest-neighbour decoder could reach more reliably by construction; the two oracle versions differ simultaneously in four link-budget terms (visibility, two-arm efficiency, latency, the rate floor), so nothing here isolates which one drove the change. The broader conclusion survives regardless: representation still matters several times more than optimiser choice, even though the specific multiplier turned out to be a property of this benchmark’s physical model rather than a universal constant. Designing the decoding map remains worth more effort than tuning or selecting the optimiser—just less dramatically so than we previously reported.

A related but separate result concerns dimensionality. The problem is formally posed with four objectives, yet the front we actually find has a single dominant principal component (96.1% of the variance), and none of the machinery built to counteract dominance resistance—reference-point niching, decomposition, hypervolume-contribution selection, reference-vector adaptation, adaptive geometry estimation—beats plain crowding-distance or archive-density selection among the top-performing algorithms. Reporting effective alongside nominal dimensionality costs little and changes which comparisons are worth taking seriously.

There is also a genuine, if largely benign, representation defect: the decision vector embeds a stochastic realisation index rather than keeping it separate. Its cost is measurable directly (Section 6.6) as a roughly 7% HV swing between the best- and worst-performing fixed constants we tested, with no way to know in advance which is which; treating the index as a free, optimised variable removes that risk, but at the cost of a search dimension the representation should not need in the first place.

Finally, the NSGA-II-vs-NSGA-III comparison turned out to be a useful diagnostic in its own right. Under the earlier, uncorrected oracle it sat close enough to the Holm threshold that merely enlarging the correction family from five to seven algorithms—with no change to the underlying data—flipped it from significant to not. Under the corrected oracle that fragility disappears: the comparison is significant by a comfortable margin under both the five- and the seven-algorithm family ($p_{\mathrm{Holm}}<0.001$ either way), and no longer depends on which family is declared. In hindsight, a result whose verdict moves with a defensible choice of correction family was itself telling us the effect was small relative to the noise floor—worth checking for, in any paper reporting a Holm-corrected comparison, before trusting the family size that happens to have been chosen.

### 7.2. Why NSGA-II Leads NSGA-III, and Why That Is a Narrower Claim than It Sounds

The 5.4% mean-HV gap in favour of NSGA-II is modest next to its 43.2% advantage over MOEA/D, but it survives Holm correction over the full seven-algorithm family under both statistical design ($p_{\mathrm{Holm}}=0.001$ unpaired, Table 5; 0.005 paired, Table 7), with a moderate-to-large effect size in NSGA-II’s favour under both (δ=+0.62, $r_{rb}=+0.74$), a clean result under the satellite-visibility-corrected oracle (Section 4.2). It is also a narrower claim than “NSGA-II is the better algorithm”: across the four additional instances, only *regional_eu_na* gives corrected evidence for the same ordering, while two of the remaining three (*metro_europe* and, now with a full 10-seed budget, *global_n200*) reverse the point estimate, neither significantly (Section 6.7). We therefore make the claim the 100-city benchmark actually supports—NSGA-II significantly outperforms NSGA-III on *this* instance, under both statistical designs, by a real but modest margin—without extending it to a general claim that NSGA-II is preferable across city distributions and scales, which the multi-instance evidence does not support. This nuance is itself consistent with previous empirical findings [72,73] that with only four objectives, the reference-point niching of NSGA-III provides limited additional diversity over NSGA-II’s crowding-distance—limited, evidently, in both directions, since neither algorithm dominates the other once the instance varies. Whether NSGA-III becomes preferable at higher *M*, or when explicit preference articulation is required [17], is outside what this four-objective benchmark can test. We report cross-seed consistency (std of HV across seeds) for each algorithm alongside the mean, and avoid recommending one algorithm as categorically “safer” without a stability metric to back that claim; where the Holm-corrected Mann–Whitney test (primary) does not reject the null hypothesis for a given pair, we report the comparison as statistically indistinguishable rather than asserting a ranking.

### 7.3. Why Dominance-And-Density Algorithms Do as Well as Anything Else on a Nominally Many-Objective Problem

The top group on this benchmark consists of NSGA-II and SPEA2—classical Pareto-dominance-and-density algorithms designed for two-to-three objectives, with no reference-direction, decomposition or hypervolume-indicator machinery—together with AGE-MOEA-II, whose adaptive geometry estimation confers no measurable advantage here. None of the algorithms whose defining mechanisms exist specifically to counteract dominance resistance in many-objective spaces (NSGA-III’s reference-point niching, MOEA/D’s decomposition, SMS-EMOA’s hypervolume-contribution selection, RVEA’s reference-vector adaptation) improves on them, and two are clearly worse. Read next to the effective-dimensionality result above (Threats to validity, item viii: PC1 explains 96.1% of the pooled evolutionary front’s variance, PC1+PC2 99.1%), a plausible reading is that dominance comparisons degrade as the number of *effectively independent* objectives grows, not as the number of *formally defined* objectives grows, and on this benchmark, the found front’s effective dimensionality is close to one or two rather than four. Under a problem this close to two-objective in practice, plain Pareto dominance would retain most of its selection pressure, so the extra machinery the other algorithms carry would have comparatively little degradation to correct for while still adding variance (SMS-EMOA, RVEA) or a coarser, decomposition-constrained search (MOEA/D). We offer this as an explanatory hypothesis consistent with the data, not as something the experiment tests directly: separating it from alternatives would require constructing instances of controlled effective dimensionality and re-running the comparison on each, which we have not done. What the experiment does establish is the pattern itself, and that it does not depend on the generation of algorithm design: AGE-MOEA-II’s adaptive geometry estimation [69] is a 2022 mechanism built to counteract dominance resistance, and on this front, it performs neither better nor worse than a 2001 archive-density method (Section 6.1).

### 7.4. Why MOEA/D Underperforms

MOEA/D’s Tchebycheff scalarisation [74] requires careful tuning of neighbourhood size and weight vectors [75]; with the default $n_{\mathrm{neighbors}}=15$ and das-dennis weights, it under-explores the front and produces far smaller unique-solution counts (6.7±2.1 vs. 89.9 and 65.1, Table 3), even at a matched evaluation budget (Section 4.3): its population repeatedly reconverges on a small handful of distinct approximate non-dominated objective vectors rather than maintaining a diverse spread across the front. This is not offset by better spacing—once per-run fronts are deduplicated by objective vector, MOEA/D shows no significant spacing advantage over NSGA-II or NSGA-III (Table 7). We did not perform a neighbourhood-size or weight-vector sweep for MOEA/D in this study: a shallow, under-powered sweep would not settle whether a fairer configuration closes the gap, and a properly powered one is a substantial undertaking in its own right, so hyperparameter tuning of MOEA/D remains explicitly left as future work rather than attempted here.

### 7.5. Why the Parametric Representation Works

The mixed parametric representation reduces the search dimension from 4950 (a fully free per-edge encoding) to 11, a four hundred-fold reduction. This places the problem squarely in the regime where evolutionary operators are designed to work (SBX/PM are derived for low-dimensional continuous spaces). The price is that the representation is no longer universal: some valid topologies are unreachable from any x∈X. However, the 561-point deduplicated combined Pareto front shows that the reachable subspace is sufficient to expose a wide range of trade-offs—though Section 6.4 shows this subspace does not dominate every classical construction, discussed further below.

### 7.6. Threats to Validity

(i) *Oracle parameters* are taken from the literature but specific values may vary by deployment. The companion paper [18] performs a scenario-based, intra-family sensitivity analysis in which the oracle’s eight physical parameters are perturbed by −40% to +33%; this yields Pearson r=0.976–0.992 between the perturbed and unperturbed oracle’s outputs on all four objectives, but mean relative differences of 9–13% on three of the four despite this high correlation. That analysis compares the same analytical formulation against itself under parameter perturbation, not against an independently implemented oracle or a discrete-event simulator, so it should be read as evidence against gross parameter-sensitivity artefacts rather than as a general robustness guarantee for the rankings reported here. (ii) *Algorithm tuning* of MOEA/D was minimal (canonical Tchebycheff scalarisation, $n_{\mathrm{neighbors}}=15$); a more extensively tuned MOEA/D (alternative decomposition, neighbourhood size sweep) could close part of the gap. NSGA-II, NSGA-III, SMS-EMOA, RVEA, SPEA2 and AGE-MOEA-II all used canonical settings and the same crossover/mutation operators; we did not perform an operator- or hyperparameter-tuning sweep for any of the seven algorithms, so the ranking in Section 6.1 should be read as a comparison under matched, canonical configurations rather than a claim that it is invariant to further tuning of any one algorithm. (iii) *Single oracle; one directional single-span fibre-loss sanity check.* We have not cross-validated the BDCZ oracle against a discrete-event quantum network simulator in any general sense. What we performed is narrower than the phrase “oracle cross-validation” would suggest and we do not use that phrase for it: a directional, single-span, fibre-only loss-vs-distance sanity check using SeQUeNCe [76] (Argonne’s Python-native discrete-event quantum network simulator, v0.8.5, installs via pip with no account required). SeQUeNCe’s QuantumChannel implements photon loss as $1-10^{-\mathrm{attenuation}\times \mathrm{distance}/10}$, structurally identical to this paper’s per-span fibre transmittance law; we ran SeQUeNCe’s own midpoint-heralded entanglement-generation protocol (EntanglementGenerationA, the same elementary-link scheme fiber_link() models) between two quantum routers at seven single-span fibre distances (1–50 km, within this paper’s 50 km repeater-spacing regime, so no swapping is needed for a fair comparison), 10 seeds each, and counted how many of 50 quantum memories per node reached successful entanglement within a fixed simulation window. The median entangled-memory count fell monotonically with distance (47.5→41.5→32.0→18.5→7.0→3.5→1.0 across 1,5,10,20,30,40,50 km, zero rank violations), in perfect rank agreement (Spearman ρ=1.0) with the analytical oracle’s own transmittance decay. This independently confirms the *direction* of the oracle’s single-span fibre distance-dependence using an unrelated simulator’s own discrete-event physics, and nothing beyond it. Explicitly outside its scope: absolute magnitudes of any quantity; the oracle’s *fidelity* model; entanglement swapping and therefore every multi-hop route; and the satellite and free-space optical media entirely, neither of which is exercised by this check at all. On fidelity specifically, SeQUeNCe’s baseline protocol fixes the produced state’s fidelity to a constant supplied by the memory (memory.raw_fidelity), rather than deriving it from channel loss or distance as fiber_link() does, so a fidelity-vs-distance cross-check would require implementing custom noise modelling beyond SeQUeNCe’s standard elementary-link protocol—left as future work alongside the multi-hop swapping validation discussed in Section 8. (iv) *Parametric representation not universal.* The 11-variable decoder cannot reach every valid topology; the reachable subspace X exposes the trade-offs observed in our 561-point deduplicated combined front, but we cannot rule out the existence of superior topologies outside the parametric family—indeed, Section 6.4 shows several classical constructions the decoder does not dominate. Quantifying this gap further (e.g., via local search around Pareto points) is left as future work. (v) *Statistical significance.* The mean-HV ordering {NSGA-II, SPEA2, AGE-MOEA-II} > NSGA-III ≳ SMS-EMOA > RVEA > MOEA/D (Table 3) is supported by a Kruskal–Wallis omnibus test across the seven algorithms (independent samples, the primary analysis) followed by Holm-corrected pairwise Mann–Whitney tests applied jointly across all 63 pairwise tests (three metrics × 21 algorithm pairs), controlling the family-wise error rate across the full test family rather than within each metric separately. Only some of that ordering is separated by the corrected tests: the top three are mutually indistinguishable from one another; NSGA-III is significantly separated from all three of them in both HV and IGD under both the unpaired and the paired design ($p_{\mathrm{Holm}}\leq 0.02$ throughout); but NSGA-III is *not* separated from SMS-EMOA in HV or IGD under either design ($p_{\mathrm{Holm}}=1.000$ and 0.085 unpaired), and the one significant difference between them, spacing under the unpaired design ($p_{\mathrm{Holm}}=0.009$, δ=−0.52, favouring NSGA-III’s more uniform front), does not survive the paired design ($p_{\mathrm{Holm}}=0.345$)—so the ≳ sign above marks a relationship that is metric- and design-dependent, not a settled ordering (Table 5). The analysis is cross-checked against a common-seed paired (Friedman omnibus, Wilcoxon pairwise) sensitivity analysis that assumes seed-index pairing across algorithms instead: the two designs agree on 60 of the 63 comparisons (disagreeing only on HV for NSGA-III vs. RVEA and on spacing for NSGA-III vs. MOEA/D and NSGA-III vs. SMS-EMOA), and agree in particular that NSGA-II vs. NSGA-III *is* significant on all three metrics under both designs (Section 6.1 reports the full breakdown). NSGA-II’s mean HV is itself driven by a subset of seeds reaching a qualitatively higher-HV region of the search space (Section 6.1 discusses this bimodality directly and reports the median alongside the mean for exactly this reason). For the decoder ablation, a Friedman omnibus test (paired by seed) followed by Holm-corrected paired Wilcoxon signed-rank comparisons against the full decoder (Section 6.5) is used analogously, since the eight ablation variants share the same 30 seeds. (vi) *Parallel media on the same city pair are decoder-visible but routing-invisible.* The decoder can store more than one medium (e.g., both fibre and satellite) for the same city pair, but the oracle’s path search collapses parallel media to the single highest-fidelity one for routing (Section 4.2); a lower-fidelity parallel link therefore contributes to the stored topology’s raw edge count without affecting any of the four objectives, a structurally neutral dimension in the search. In practice, this is a modest effect on the released front: the mean raw medium-specific edge count (808) exceeds the mean effective, oracle-routed edge count (782) by about 3.3% (Table 8), so parallel media are present but not a dominant source of the gap between 561 unique objective vectors and 944 unique topologies—most of that gap instead reflects genuinely different edge sets that happen to reach numerically identical objective values. (vii) *The decoder’s seed offset (σ) is optimised as an eleventh decision variable, but it is a pseudorandom-number-generator seed, not a physical design parameter*: it selects which realisation of the decoder’s stochastic edge-sampling process is decoded, and two numerically close seed values need not produce similar topologies, in some tension with the locality SBX/polynomial mutation are designed to exploit. Section 6.6 reports a direct ablation of this concern across three independently tested fixed constants: two of the three are statistically indistinguishable from optimising σ ($p_{\mathrm{Holm}}=1.000$ against σ=42 and σ=500), while the third is significantly *worse* than optimising (σ=0, $p_{\mathrm{Holm}}=0.015$), and the fixed constants are significantly different from *each other* (σ=0 vs. σ=42 or σ=500, $p_{\mathrm{Holm}}\leq 0.035$, |δ|≥0.58), while averaging the fitness over multiple realisations per candidate is significantly *worse* than every other variant tested. Optimising σ is therefore not demonstrated to beat the best possible fixed value, but it matches the good fixed constants and clearly avoids the risk of landing on a bad one with no a priori way to tell which is favourable. We are explicit about what this ablation does *not* settle. It shows that the constants we happened to test that are good are statistically indistinguishable from searching over σ, and that the one we tested that is bad is detectably worse than searching; it does not establish that treating a realisation index as a continuous decision variable is methodologically sound, and the argument that it is not—neighbouring values of σ decode to unrelated graphs, which is the opposite of the local structure SBX and polynomial mutation exploit—stands unrefuted. The clean design would separate the ten physical design parameters from the choice of realisation: a deterministic decoder, common random numbers across candidates, or a robust fitness evaluated over several realisations at a budget matched in oracle calls rather than in generations (variant C above was matched on generations, and so paid 3× the oracle cost). We did not adopt it here because doing so would require re-running every other experiment in this paper for consistency, and we report the finding as a validated recommendation for future work on this decoder rather than as a change we have made. (viii) *Effective dimensionality of the objective space.* The pairwise correlation structure on the pooled evolutionary front (Section 6.2) shows fidelity, rate and latency are all strongly mutually aligned (|ρ|≥0.89), while coverage is the objective genuinely in tension with the other three (|ρ|≥0.92); the search is nominally four-objective but its found front may behave closer to a two-cluster (coverage-vs.-the-rest) trade-off than to four independently conflicting dimensions. A standardised PCA on the 561-point global front confirms this directly: the first principal component alone explains 96.1% of the variance, and the first two components 99.1% (Script: analyze_effective_dimensionality.py, released with the code); the first component loads all four objectives with comparable magnitude and a sign pattern consistent with the alignment/tension structure above (fidelity and rate loading opposite coverage, given the minimise/maximise conventions). This does not change that four distinct, physically meaningful quantities are being jointly optimised and reported, but it does mean the found front’s effective degrees of freedom are closer to one or two than to four, and we treat that as a property of the benchmark rather than describing it throughout as four independently conflicting dimensions.

The low effective dimensionality could in principle be an artefact of measuring all four objectives along the same fidelity-optimal path, rather than each objective along its own best route, which would correlate fidelity, rate and latency almost by construction. We tested this: re-decoding every pooled-front point and recomputing all four objectives under two alternative routing policies (rate-optimal: maximise the bottleneck per-hop rate; latency-optimal: minimise summed latency, both using the same exact search machinery as the canonical fidelity-optimal policy, just a different edge-composition rule) (Script: routing_policy_sensitivity_v3.py). PC1 remains essentially unchanged across all three policies (96.1% fidelity-optimal, 94.5% rate-optimal, 96.3% latency-optimal): the four objectives remain strongly aligned regardless of which one the path search is optimised for. The scope of this check is worth stating precisely. It re-evaluates the 561 points *discovered* under fidelity-optimal routing; it does not re-run the optimisation under the alternative policies. What it establishes is that the canonical front stays low-dimensional when re-scored, not that a front obtained by optimising from scratch under rate- or latency-optimal routing would have the same dimensionality. Testing the stronger claim would require repeating at least part of the search under each policy.

## 8. Conclusions and Future Work

We have presented, to the best of our knowledge, the first many-objective evolutionary topology-design framework over a heterogeneous, intercontinental candidate space for the quantum internet—a narrower claim than topology design for quantum networks in general (Section 2 distinguishes it from prior single-objective and fixed-topology formulations)—with four physically motivated objectives: fidelity, rate, latency and coverage, across heterogeneous fibre/satellite/FSO infrastructure. An 11-variable mixed parametric representation, evaluated by an analytical BDCZ oracle whose path selection is exact under the stated Werner-composition model and whose satellite links respect a single-satellite visibility limit, makes a problem that degenerates under direct per-edge categorical optimisation tractable with standard evolutionary algorithms on commodity CPU hardware, producing a 561-point deduplicated non-dominated front in minutes per run.

Four results are robust to the statistical and framing choices we were able to test. *(i) The representation, not the optimiser, is what makes the problem tractable*: a fully categorical edge-and-medium baseline reaches only a fifth of the parametric decoder’s hypervolume at this scale, while every optimiser tested succeeds on the parametric decoder, and random search over the same bounds already reaches 92% of NSGA-II’s hypervolume on a shared scale—most of the decoder’s advantage over categorical search comes from the representation itself, not from evolutionary selection pressure on top of it. *(ii) Seven algorithms spanning 2001–2022 separate into a top group, a middle tier and a tail*: NSGA-II, SPEA2 and AGE-MOEA-II are mutually indistinguishable on HV, IGD and spacing after Holm correction over the full 63-test family, and each significantly outperforms NSGA-III, SMS-EMOA, RVEA and MOEA/D on HV; NSGA-III and SMS-EMOA remain statistically indistinguishable on HV and IGD (the primary design favours NSGA-III on spacing, unconfirmed by the paired design) and sit between the top group and RVEA/MOEA/D, which are clearly behind. Mechanisms designed to counteract dominance resistance buy nothing measurable for the top-performing algorithms on a front whose first principal component explains 96.1% of the variance. *(iii) Under NSGA-III, at the tested budget, oracle and parameter ranges, fibre is necessary and satellite is not decisive*: every fibre-free ablation variant collapses hypervolume ($p_{\mathrm{Holm}}<1.4\times 10^{-8}$), while satellite’s contribution beyond fibre alone and the inter-region bonus’s contribution are both statistically indistinguishable from zero, and fibre+FSO without satellite is significantly *higher* than the full decoder ($p_{\mathrm{Holm}}=0.003$) for reasons this study does not establish. The ablation was run with a single algorithm (NSGA-III), now the one significantly separated from the top group on the main comparison (finding (ii) above), so this medium-necessity ordering is established for that algorithm and has not been confirmed to hold for NSGA-II, SPEA2 or AGE-MOEA-II. *(iv) The evolutionary front dominates classical design almost entirely*: of 14 classical constructors tested, only a random geometric graph at a 5000 km distance threshold remains non-dominated, by a narrow coverage margin; every hub-and-spoke variant, which was the non-dominated family before the satellite model was corrected, is now dominated.

Two limitations bound how the released front should be used. NSGA-II’s mean-HV lead over NSGA-III is bimodal across seeds rather than uniform, and, although this lead now survives Holm correction on the main 100-city instance under both statistical designs, it reverses in point estimate (without reaching significance) on two of the four additional instances tested. And **no** front point connects all 100 cities under a single-satellite visibility limit; the median point reaches 50.9% of city pairs, so the front spans a coverage axis from near-global designs to sparser, higher-fidelity solutions and should be filtered by connectivity or minimum coverage before use (Section 6.2).

The oracle’s external validation remains narrow: a directional, single-span, fibre-only loss-vs-distance check against SeQUeNCe [76] (Spearman ρ=1.0 across seven distances), which says nothing about fidelity, swapping, satellite or FSO. Closing that gap is the highest-priority next step: a NetSquid [77] or SeQUeNCe/QuISP [78] multi-hop entanglement-swapping simulation whose protocol assumptions are verifiably equivalent to this paper’s Werner-composition BDCZ model. Beyond it: an explicit connectivity constraint or coverage-conditioned fronts, motivated directly by the connectivity limitation noted above; a decoder that separates its ten physical parameters from the realisation index rather than optimising over the latter (Section 6.6); a parsimonious reduced-variable decoder; hyperparameter tuning of MOEA/D; a fifth objective (infrastructure cost) once deployment-grade cost models exist; a medium-necessity ablation on the denser *metro_europe* instance to test whether FSO’s marginal contribution is an artefact of sparse global-city geography; and a multi-satellite constellation model, which this single-satellite benchmark cannot speak to and which Section 6.2 identifies as the most direct route to recovering fully connected solutions on the Pareto front.   

## Figures and Tables

**Table 1:** Physical parameters of the analytical BDCZ oracle.

Parameter	Symbol	Value
**Fibre**		
Wavelength	$\lambda_{f}$	1550 nm
Attenuation	$\alpha_{f}$	0.20 dB/km
Repeater spacing	$L_{\mathrm{rep}}$	50 km
Base fidelity	$F_{0}^{f}$	0.99
Decoherence rate	$\gamma_{f}$	$1.5\times 10^{-4}$ km^−1^
Detector efficiency	$\eta_{f}$	0.85
Source rate	$r_{f}$	$10^{7}$ Hz
Swap success	$p_{\mathrm{swap}}$	0.50
**Satellite (LEO)**		
Altitude	*h*	500 km
Tx aperture	$D_{tx}$	0.30 m
Rx aperture	$D_{rx}$	1.00 m
Wavelength	λ	810 nm
Atm. transmittance	$\tau_{\mathrm{atm}}$	0.80
Pointing loss	$L_{p}$	3.0 dB
Duty cycle	δ	0.005
**Satellite (LEO)**		
Base fidelity	$F_{0}^{s}$	0.83
Decoherence rate	$\gamma_{s}$	$5\times 10^{-6}$ km^−1^
Detector efficiency	$\eta_{s}$	0.50
Source rate	$r_{s}$	$5.9\times 10^{6}$ Hz
**Free-space optical**		
Wavelength	$\lambda_{g}$	810 nm
Extinction	$\alpha_{g}$	0.50 dB/km
Base fidelity	$F_{0}^{g}$	0.92
Decoherence rate	$\gamma_{g}$	$10^{-4}$ km^−1^
Detector efficiency	$\eta_{g}$	0.70
Source rate	$r_{g}$	$10^{7}$ Hz
Max range	$d_{g}^{\max}$	150 km

**Table 2:** The eleven decoder variables and their search ranges. Four variables (marked “integer”) are rounded to the nearest integer before decoding. Satellite candidates are additionally restricted to $d_{uv}\geq 500$ km regardless of $d_{s}^{\max}$ (Algorithm 1). The upper bound on $d_{s}^{\max}$ is the single-satellite visibility limit of Equation (A6), not a free choice: beyond 4891 km, no satellite at h=500 km can see both ground stations, so the search space must not contain such links (Appendix A.2). The same principle bounds $d_{g}^{\max}$ at the FSO model’s own 150 km hard limit.

#	Parameter	Range
1	$k_{f}$ (fibre *k*-NN, integer)	[3,25]
2	$k_{s}$ (satellite *k*-NN, integer)	[2,25]
3	$k_{g}$ (FSO *k*-NN, integer)	[0,10]
4	$d_{f}^{\max}$ (max fibre dist. km)	[300,3000]
5	$d_{s}^{\max}$ (max sat. dist. km)	[1000,4891]
6	$d_{g}^{\max}$ (max FSO dist. km)	[10,150]
7	$p_{f}$ (fibre incl. prob.)	[0.10,1.0]
8	$p_{s}$ (satellite incl. prob.)	[0.10,1.0]
9	$p_{g}$ (FSO incl. prob.)	[0.10,1.0]
10	$\beta_{r}$ (inter-region bonus)	[0.0,1.0]
11	σ (seed offset, integer)	[0,999]

**Table 3:** Algorithm comparison on the quantum network topology optimisation problem (30 seeds, mean ± sample-std, canonical oracle, single shared reference front and normalisation across all seven algorithms). $|F^{★}|$ is the number of *unique* objective vectors per run after deduplication (Section 6.1), not the raw non-dominated population size. Bold marks the best value per column. Rows are ordered by mean HV; the top three are not separated by the corrected tests (see the pairwise tests in Section 6.1). Arrows indicate the direction of improvement: ↑ higher is better, ↓ lower is better.

Algorithm	HV ↑	IGD ↓	Spacing ↓	$|F^{★}|$	Time (min)
NSGA-II	0.353±0.040	0.063±0.024	0.018±0.005	89.9±3.5	4.9±1.0
SPEA2	0.352±0.038	0.063±0.024	0.020±0.005	103.8±0.9	3.2±0.3
AGE-MOEA-II	0.347±0.037	0.068±0.022	0.018±0.005	94.3±3.0	3.0±0.3
NSGA-III	0.335±0.037	0.072±0.022	0.033±0.030	65.1±6.3	4.6±0.9
SMS-EMOA	0.327±0.027	0.075±0.021	0.034±0.010	81.5±6.0	6.4±1.4
RVEA	0.316±0.030	0.151±0.092	0.262±0.063	9.7±1.6	3.2±1.1
MOEA/D	0.247±0.010	0.603±0.018	0.053±0.037	6.7±2.1	2.4±0.9

**Table 4:** Kruskal–Wallis omnibus test across the seven algorithms (independent samples, n=30 per algorithm), the primary statistical test used in this paper: it treats each algorithm’s 30 runs as an independent sample rather than assuming seed-index pairing across algorithms, run before any pairwise comparison to guard against fishing for a significant pair.

Metric	*H*	*p*
HV	116.327	<$10^{-6}$
IGD	137.395	<$10^{-6}$
Spacing	131.450	<$10^{-6}$

**Table 5:** Pairwise Mann–Whitney *U* tests (n=30 per algorithm, two-sided), Holm-corrected jointly across all 63 tests (3 metrics × 21 algorithm pairs), with Cliff’s δ effect size (equivalently, Vargha–Delaney $A_{12}=\left(\delta +1\right)/2$). Significance codes: ** $p_{\mathrm{Holm}}<0.01$, * $p_{\mathrm{Holm}}<0.05$.

Comparison	HV $\left(p_{\mathrm{Holm}},\delta \right)$	IGD $\left(p_{\mathrm{Holm}},\delta \right)$	Spacing $\left(p_{\mathrm{Holm}},\delta \right)$
NSGA-II vs. SPEA2	(1.000,+0.02)	(1.000,+0.14)	(1.000,−0.17)
NSGA-II vs. AGE-MOEA-II	(1.000,+0.03)	(1.000,−0.22)	(1.000,+0.04)
NSGA-II vs. NSGA-III	(0.001 **, +0.62)	(<0.001 **, −0.63)	(0.003 **, −0.58)
NSGA-II vs. SMS-EMOA	(<0.001 **, +0.66)	(0.002 **, −0.60)	(<0.001 **, −0.89)
NSGA-II vs. RVEA	(0.002 **, +0.60)	(<0.001 **, −0.86)	(<0.001 **, −1.00)
NSGA-II vs. MOEA/D	(<0.001 **, +1.00)	(<0.001 **, −1.00)	(<0.001 **, −0.70)
SPEA2 vs. AGE-MOEA-II	(1.000,+0.03)	(0.215,−0.35)	(1.000,+0.19)
SPEA2 vs. NSGA-III	(<0.001 **, +0.63)	(<0.001 **, −0.64)	(0.085,−0.41)
SPEA2 vs. SMS-EMOA	(<0.001 **, +0.66)	(0.002 **, −0.60)	(<0.001 **, −0.84)
SPEA2 vs. RVEA	(0.002 **, +0.59)	(<0.001 **, −0.85)	(<0.001 **, −1.00)
SPEA2 vs. MOEA/D	(<0.001 **, +1.00)	(<0.001 **, −1.00)	(<0.001 **, −0.69)
AGE-MOEA-II vs. NSGA-III	(0.002 **, +0.60)	(0.002 **, −0.59)	(0.001 **, −0.62)
AGE-MOEA-II vs. SMS-EMOA	(<0.001 **, +0.63)	(0.005 **, −0.55)	(<0.001 **, −0.87)
AGE-MOEA-II vs. RVEA	(0.005 **, +0.55)	(<0.001 **, −0.83)	(<0.001 **, −1.00)
AGE-MOEA-II vs. MOEA/D	(<0.001 **, +1.00)	(<0.001 **, −1.00)	(<0.001 **, −0.70)
NSGA-III vs. SMS-EMOA	(1.000,+0.21)	(0.085,−0.41)	(0.009 **, −0.52)
NSGA-III vs. RVEA	(0.013 *, +0.50)	(<0.001 **, −0.84)	(<0.001 **, −0.99)
NSGA-III vs. MOEA/D	(<0.001 **, +1.00)	(<0.001 **, −1.00)	(0.014 *, −0.50)
SMS-EMOA vs. RVEA	(0.085,+0.41)	(<0.001 **, −0.84)	(<0.001 **, −1.00)
SMS-EMOA vs. MOEA/D	(<0.001 **, +1.00)	(<0.001 **, −1.00)	(0.113,−0.39)
RVEA vs. MOEA/D	(<0.001 **, +1.00)	(<0.001 **, −1.00)	(<0.001 **, +0.98)

**Table 6:** Friedman omnibus test across the seven algorithms (paired by seed, n=30)—common-seed sensitivity check, not the primary test (see Table 4).

Metric	$\chi^{2}$	*p*
HV	126.000	<$10^{-6}$
IGD	135.186	<$10^{-6}$
Spacing	115.700	<$10^{-6}$

**Table 7:** Pairwise Wilcoxon signed-rank tests (n=30, two-sided), Holm-corrected jointly across all 63 tests (3 metrics × 21 algorithm pairs), with matched-pairs rank-biserial correlation $r_{rb}$—common-seed sensitivity check, not the primary test (see Table 5). Significance codes: ** $p_{\mathrm{Holm}}<0.01$, * $p_{\mathrm{Holm}}<0.05$. With 30 paired seeds the two-sided Wilcoxon signed-rank test’s own minimum achievable *p*-value is $2/2^{30}\approx 1.9\times 10^{-9}$, far below every *p*-value reported below, so none of these results sit at the test’s resolution floor; at a smaller paired sample, this floor can bind and needs checking explicitly rather than assumed away.

Comparison	HV $\left(p_{\mathrm{Holm}},r_{\mathrm{rb}}\right)$	IGD $\left(p_{\mathrm{Holm}},r_{\mathrm{rb}}\right)$	Spacing $\left(p_{\mathrm{Holm}},r_{\mathrm{rb}}\right)$
NSGA-II vs. SPEA2	(1.000,+0.25)	(1.000,−0.03)	(1.000,−0.18)
NSGA-II vs. AGE-MOEA-II	(1.000,+0.31)	(0.839,−0.34)	(1.000,+0.02)
NSGA-II vs. NSGA-III	(0.005 **, +0.74)	(0.020 *, −0.67)	(0.023 *, −0.66)
NSGA-II vs. SMS-EMOA	(<0.001 **, +0.82)	(0.002 **, −0.77)	(<0.001 **, −1.00)
NSGA-II vs. RVEA	(0.004 **, +0.75)	(<0.001 **, −0.89)	(<0.001 **, −1.00)
NSGA-II vs. MOEA/D	(<0.001 **, +1.00)	(<0.001 **, −1.00)	(<0.001 **, −0.88)
SPEA2 vs. AGE-MOEA-II	(1.000,+0.04)	(0.281,−0.47)	(1.000,+0.21)
SPEA2 vs. NSGA-III	(0.007 **, +0.72)	(0.020 *, −0.66)	(0.099,−0.56)
SPEA2 vs. SMS-EMOA	(0.002 **, +0.79)	(0.013 *, −0.69)	(<0.001 **, −0.96)
SPEA2 vs. RVEA	(0.020 *, +0.67)	(<0.001 **, −0.92)	(<0.001 **, −1.00)
SPEA2 vs. MOEA/D	(<0.001 **, +1.00)	(<0.001 **, −1.00)	(<0.001 **, −0.85)
AGE-MOEA-II vs. NSGA-III	(0.019 *, +0.67)	(0.033 *, −0.63)	(0.006 **, −0.73)
AGE-MOEA-II vs. SMS-EMOA	(0.001 **, +0.79)	(0.005 **, −0.74)	(<0.001 **, −1.00)
AGE-MOEA-II vs. RVEA	(0.034 *, +0.63)	(<0.001 **, −0.88)	(<0.001 **, −1.00)
AGE-MOEA-II vs. MOEA/D	(<0.001 **, +1.00)	(<0.001 **, −1.00)	(<0.001 **, −0.89)
NSGA-III vs. SMS-EMOA	(0.244,+0.49)	(0.289,−0.46)	(0.345,−0.44)
NSGA-III vs. RVEA	(0.092,+0.57)	(0.002 **, −0.78)	(<0.001 **, −1.00)
NSGA-III vs. MOEA/D	(<0.001 **, +1.00)	(<0.001 **, −1.00)	(0.159,−0.53)
SMS-EMOA vs. RVEA	(0.470,+0.41)	(<0.001 **, −0.85)	(<0.001 **, −1.00)
SMS-EMOA vs. MOEA/D	(<0.001 **, +1.00)	(<0.001 **, −1.00)	(0.271,−0.48)
RVEA vs. MOEA/D	(<0.001 **, +1.00)	(<0.001 **, −1.00)	(<0.001 **, +1.00)

**Table 8:** Resource use across the pooled evolutionary front: number of edges and fraction by medium (across all 561 deduplicated solutions). |E| is reported three ways since a topology can store more than one medium per city pair (Section 6.2): raw medium-specific edges (a pair with 2 media counts as 2), unique city pairs connected by *any* medium, and the edges the oracle actually routes over after collapsing parallel media to the highest-fidelity one (identical to the unique-pairs count by construction). Classical baselines have exactly one medium per pair, so their |E| in the classical-baseline tableis directly comparable to the unique-pairs/effective-oracle columns here, not the raw medium-specific one. Medium fractions are reported both pooled (sum over all edges in all topologies—implicitly weights larger topologies more) and as the mean ± std of each topology’s own fraction (every topology weighted equally); the two differ materially and answer different questions.

|E| Definition	Min	Median	Mean	Max
Raw medium-specific	37	1049	808	1765
Unique city pairs	36	971	782	1221
Effective (oracle routing)	36	971	782	1221
		**Fibre**	**Satellite**	**FSO**
Pooled fraction of edges		12.4%	87.1%	0.5%
Per-topology fraction (mean ± std)		25.3±25.4%	73.5±26.6%	1.2±1.9%

**Table 9:** Results of 4950-variable categorical baseline vs. 11-variable parametric decoder (30 seeds, identical budget: pop 104, 80 generations). HV/IGD computed against a single, deduplicated reference front shared by all four rows; $|F^{★}|$ is each row’s own deduplicated unique-objective-vector count (Section 6.1). These HV/IGD values are *not* directly comparable to Table 3: that table’s seven-algorithm reference front and this table’s four-row, decoder-plus-categorical reference front are pooled from different runs and normalised independently, so, e.g., NSGA-II’s decoder HV here (0.662) differs from its value in Table 3 (0.353) despite referring to the same underlying runs.

Representation and Algorithm	HV	IGD	$|F^{★}|$	Time (min)
11-var. decoder + NSGA-II	0.662±0.046	0.043±0.016	89.9±3.5	4.87±1.00
11-var. decoder + NSGA-III	0.640±0.042	0.053±0.015	65.1±6.3	4.56±0.94
4950-var. categorical + NSGA-II	0.125±0.022	0.666±0.021	64.6±16.5	0.71±0.14
4950-var. categorical + NSGA-III	0.124±0.018	0.678±0.018	10.1±2.1	0.63±0.13

**Table 10:** Classical network-design baselines (heterogeneous medium, canonical oracle) vs. the pooled evolutionary front: all 14 constructors referenced in Section 6.4. $\bar{F}$: fidelity; ${\bar{R}}_{\log}$: log-rate; $\bar{L}$: latency (s); $C_{\tau }$: coverage. †: non-dominated by the pooled evolutionary front (i.e., not beaten by any evolutionary solution on all four objectives simultaneously). The final row reports the *range* spanned by the 561-point Pareto front, not a single solution’s values—no individual Pareto-optimal topology need actually attain the elementwise mean or midpoint of these ranges, and 13/14 classical constructors above are dominated by *some* point on this front (Section 6.4), not necessarily by any one representative point.

Method	|E|	$\bar{F}$	${\bar{R}}_{\log}$	$\bar{L}$	$C_{\tau }$
MST	99	0.354	1.155	0.099	0.048
*k*-NN (k=3)	197	0.452	2.723	0.079	0.072
*k*-NN (k=5)	330	0.414	2.164	0.118	0.104
*k*-NN (k=7)	479	0.442	2.473	0.105	0.135
*k*-NN (k=10)	668	0.461	2.621	0.092	0.162
RGG (500 km)	85	0.736	7.328	0.010	0.036
RGG (1000 km)	280	0.726	6.644	0.011	0.077
RGG (2000 km)	658	0.707	6.415	0.021	0.144
RGG (3000 km)	910	0.638	5.140	0.041	0.191
RGG (5000 km) ^†^	1356	0.632	3.632	0.062	0.278
Hub-and-spoke (5 hubs)	485	0.353	1.075	0.105	0.117
Hub-and-spoke (10 hubs)	945	0.432	1.826	0.092	0.146
Hub-and-spoke (15 hubs)	1380	0.539	2.842	0.076	0.177
Region-aware MST	105	0.362	1.170	0.092	0.049
**Pooled evolutionary front (range)**	36–1221	0.612–0.866	3.52–9.70	0.004–0.063	0.013–0.263

**Table 11:** Decoder ablation: eight variants evaluated with NSGA-III (30 seeds, mean ± sample std, canonical oracle). Variant labels A–E, G–I follow this study’s internal design naming; F was never assigned to a final variant and is not a missing/omitted row. $|F^{★}|$ is the deduplicated unique-objective-vector count per run, not the raw non-dominated population size (Section 6.5). IGD is measured against variant A’s own pooled front (so A’s value is close to zero by construction); IGD_union_ is the genuinely symmetric alternative, measured per-seed against the shared union of all eight variants’ pooled fronts and reported as mean ± sample std like every other column. Variant I’s HV std of 0.0000 is not a rounding artefact of a constant value: its 30 per-seed HV values genuinely span only 0.096810–0.096839 (four significant figures beyond the displayed precision), consistent with FSO-only leaving only 11 of 4950 city pairs reachable at all, so NSGA-III converges to essentially the same near-optimal front regardless of seed.

Variant	HV ↑	IGD ↓	IGD_union_ ↓	Spacing ↓	$|F^{★}|$
A. Full decoder	0.1922±0.0188	0.0344±0.0091	0.2282±0.0486	0.0259±0.0228	65.1±6.3
B. Fibre only	0.2011±0.0119	0.6327±0.0013	0.4142±0.0026	0.0288±0.0073	49.6±4.1
C. Fibre + satellite	0.1926±0.0200	0.0348±0.0105	0.2270±0.0493	0.0249±0.0206	62.4±8.4
D. Fibre + FSO	0.2055±0.0082	0.6339±0.0022	0.3955±0.0059	0.0284±0.0057	62.3±6.0
E. No inter-region bonus	0.1935±0.0194	0.0348±0.0114	0.2246±0.0516	0.0233±0.0145	65.6±7.0
G. Satellite only	0.1416±0.0068	0.1207±0.0086	0.2290±0.0351	0.0553±0.0376	50.8±6.0
H. Satellite + FSO	0.1644±0.0181	0.1106±0.0179	0.1855±0.0636	0.0381±0.0245	67.1±7.1
I. FSO only	0.0968±0.0000	1.0409±0.0001	0.6740±0.0001	0.0159±0.0007	10.3±0.4

**Table 12:** Sigma ablation (NSGA-III, 15 seeds, mean ± sample std, canonical oracle, own block-specific normalisation—not comparable to Table 3). Variants B are genuinely 10-variable Problems (not an 11-variable Problem with a collapsed bound); σ is padded on only immediately before the oracle call.

Variant	HV ↑	IGD ↓	$|F^{★}|$
A. σ optimised (11-var.)	0.447±0.051	0.046±0.011	65.0±6.1
B. σ fixed at 0 (10-var.)	0.422±0.022	0.050±0.007	66.9±5.8
B. σ fixed at 42 (10-var.)	0.439±0.039	0.047±0.010	61.1±7.3
B. σ fixed at 500 (10-var.)	0.451±0.053	0.045±0.012	62.3±5.2
C. σ averaged, K=3 (10-var.)	0.412±0.003	0.055±0.001	74.2±5.2

## Data Availability

The pooled evolutionary non-dominated front (union of all seven algorithms—NSGA-II, NSGA-III, MOEA/D, SMS-EMOA, RVEA, SPEA2 and AGE-MOEA-II—across 30 seeds each, tagged by source algorithm: 561 unique objective vectors, 944 structurally distinct topologies, with full per-point algorithm/seed provenance), the analytical BDCZ oracle implementation, the decoder, all experimental scripts and the JSON results files are released as an open, permanently archived reproducibility package, QI-Bench-Pareto-v1 (code under MIT, data under CC-BY-4.0), at https://github.com/jesusgilru-prog/qi-bench-pareto and permanently archived on Zenodo: concept DOI 10.5281/zenodo.22873153 (always resolves to the latest version) and version DOI 10.5281/zenodo.22873154 (release v1.1.0, the version this article reports).

## Appendix A. Complete Link-Level Oracle Model

This appendix states every equation the oracle uses to turn a city pair and a medium into a fidelity, a rate and a latency, so that the oracle can be audited from the paper alone, without reading the code or the companion benchmark. Numerical values of all constants are in Table 1; symbols are repeated here only where needed for the equations. All distances are great-circle (haversine, Earth radius R=6371 km) between city coordinates; all rates are in entangled pairs per second and all latencies in seconds.

### Appendix A.1. Fibre Links

A fibre link of length *d* (km) is divided into $n=\max(1,\left\lceil d/L_{\mathrm{sp}}\right\rceil )$ equal spans of length l=d/n, with $L_{\mathrm{sp}}=50$ km. Per span, the loss in dB is αl and the heralding efficiency is

(A1) $$\eta_{\mathrm{sp}}=10^{-\alpha l/10}\;\eta_{\det}^{f},\;F_{\mathrm{sp}}=F_{0}^{f}\;e^{-\gamma_{f}l},$$

with attenuation α=0.20 dB/km, detector efficiency $\eta_{\det}^{f}=0.85$, base fidelity $F_{0}^{f}=0.99$ and decoherence rate $\gamma_{f}=1.5\times 10^{-4}$ km^−1^. The *n* spans are composed by the same Werner rule used for end-to-end paths, applied n−1 times:

(A2) $$F^{\left(k+1\right)}=F^{\left(k\right)}F_{\mathrm{sp}}+\frac{1}{3}\left(1-F^{\left(k\right)}\right)\left(1-F_{\mathrm{sp}}\right),\;F^{\left(1\right)}=F_{\mathrm{sp}},$$

so $F_{\mathrm{link}}=F^{\left(n\right)}$. The rate is the source rate reduced by per-span efficiency and by the n−1 swaps needed to join the spans,

(A3) $$R_{\mathrm{link}}=R_{0}^{f}\;\eta_{\mathrm{sp}}\;p_{\mathrm{swap}}^{\;n-1},\;R_{0}^{f}=10^{7}\;s^{-1},\;p_{\mathrm{swap}}=0.50.$$

Latency is the round-trip propagation time over the total distance at the in-fibre group velocity $c_{f}=2\times 10^{8}$ m/s,

(A4) $$L_{\mathrm{link}}=\frac{2\;d\times 10^{3}}{c_{f}},$$

which is independent of *n* at fixed *d*: summing round-trip propagation over *n* spans of length d/n returns $2d/c_{f}$. This is the corrected form discussed in Section 4.2; the companion benchmark [18] multiplied Equation (A4) by a further factor *n*, making latency quadratic in distance at fixed repeater spacing.

### Appendix A.2. Satellite Links

A satellite link between two ground stations separated by a great-circle distance *d* km is modelled as a single entangled-pair source on a satellite at altitude h=500 km over the midpoint of the arc, sending one photon down each of two arms. Writing the central half-angle subtended at the Earth’s centre by each arm as φ=d/(2R) with R=6371 km, the slant range from the satellite to each station follows from the law of cosines on the triangle (Earth centre, station, satellite):

(A5) $$s=\sqrt{R^{2}+{\left(R+h\right)}^{2}-2R\left(R+h\right)\cos\varphi }\;\left(\mathrm{km}\right),$$

equal for both arms by the midpoint construction.

Visibility limit

A single satellite can serve both stations only while both lie above its horizon. At zero elevation, the station–satellite line is tangent to the Earth, giving a maximum half-angle $\varphi_{\max}=\arccos\;\left(R/(R+h)\right)$ and hence a maximum ground separation

(A6) $$d_{\max}^{s}=2R\arccos\;\frac{R}{R+h}=4891\;\mathrm{km}\;\mathrm{at}\;h=500\;\mathrm{km}.$$

Beyond $d_{\max}^{s}$, the Earth blocks the line of sight, the link does not exist, and the oracle reports $\left(F,R,L\right)=\left(0,0,10^{6}\right)$, excluding it from the path-search graph exactly as it does for out-of-range FSO links. The decoder’s upper bound on $d_{s}^{\max}$ is set to $d_{\max}^{s}$ so the search cannot propose such links (Table 2). Earlier versions of this oracle used the flat-Earth approximation $s=\sqrt{{\left(d/2\right)}^{2}+h^{2}}$ with no visibility constraint and a bound of 15,000 km; Section 4.2 records what that changed.

Link budget

With transmit aperture $D_{\mathrm{tx}}=0.30$ m, receive aperture $D_{\mathrm{rx}}=1.00$ m and wavelength λ=810 nm, the diffraction-limited divergence and spot size at the receiver are

(A7) $$\theta =\frac{\lambda }{\pi D_{\mathrm{tx}}},\;w=\theta \;s\times 10^{3}\;\left(m\right),$$

giving a geometric collection efficiency $\eta_{\mathrm{geo}}=\min\;\left(1,{\left(D_{\mathrm{rx}}/w\right)}^{2}\right)$ per arm. Every loss term above is a per-arm quantity, so the single-arm efficiency and the coincidence efficiency are

(A8) $$\eta_{\mathrm{arm}}^{s}=\eta_{\mathrm{geo}}\;T_{\mathrm{atm}}\;10^{-\Lambda_{\mathrm{point}}/10}\;\eta_{\det}^{s},\;\eta_{\mathrm{tot}}^{s}={\left(\eta_{\mathrm{arm}}^{s}\right)}^{2},$$

with $T_{\mathrm{atm}}=0.80$, $\Lambda_{\mathrm{point}}=3.0$ dB and $\eta_{\det}^{s}=0.50$. The square is not cosmetic: heralding an entangled pair requires a detection at *both* stations, so the pair efficiency is the product over the two arms. Fidelity decays with ground distance from a Micius-calibrated base [24,28], and the rate is further reduced by the fraction of time the satellite is usable:

(A9) $$F_{\mathrm{link}}=F_{0}^{s}e^{-\gamma_{s}d},\;R_{\mathrm{link}}=R_{0}^{s}\;\eta_{\mathrm{tot}}^{s}\;\delta ,$$

with $F_{0}^{s}=0.83$, $\gamma_{s}=5\times 10^{-6}$ km^−1^, $R_{0}^{s}=5.9\times 10^{6}$ s^−1^ and duty cycle δ=0.005. Both photons are emitted simultaneously and the two arms are equal by Equation (A5), so the pair is delivered after a single transit time s/c, not the sum of both arms’ transits; to this, we add the classical confirmation channel between the ground stations, assumed routed over terrestrial fibre:

(A10) $$L_{\mathrm{link}}=\frac{s\times 10^{3}}{c}+\frac{d\times 10^{3}}{c_{f}},\;c=3\times 10^{8}\;m/s.$$

### Appendix A.3. Free-Space Optical Links

FSO links are terrestrial and hard-limited to $d_{\max}^{g}=150$ km; beyond it, the link is not physical and the oracle returns $\left(F,R,L\right)=\left(0,0,10^{6}\right)$, which excludes it from the path-search graph entirely. Within range, with extinction κ=0.50 dB/km, detector efficiency $\eta_{\det}^{g}=0.70$, base fidelity $F_{0}^{g}=0.92$, decoherence $\gamma_{g}=10^{-4}$ km^−1^ and $R_{0}^{g}=10^{7}$ s^−1^:

(A11) $$\eta^{g}=10^{-\kappa d/10}\eta_{\det}^{g},\;F_{\mathrm{link}}=F_{0}^{g}e^{-\gamma_{g}d},\;R_{\mathrm{link}}=R_{0}^{g}\eta^{g},\;L_{\mathrm{link}}=\frac{2\;d\times 10^{3}}{c}.$$

The decoder’s upper bound on $d_{g}^{\max}$ is set to 150 km so the search cannot propose FSO links the model treats as invalid.

### Appendix A.4. Numerical Conventions and Edge Cases

Link tables are precomputed once for all (1002) pairs and all three media in IEEE-754 double precision, with no rounding applied at any stage; the values reported in the manuscript are rounded only for display. A few conventions are worth stating explicitly because they affect results rather than presentation. A link with $F_{\mathrm{link}}\leq 0$ is removed from the path-search graph outright, not merely down-weighted; this is what excludes out-of-range FSO edges. Where more than one medium connects the same city pair, path search keeps only the higher-fidelity one, while the lower-fidelity parallel edge stays in the stored topology and in the raw edge count (Table 8). Per-hop rates enter the bottleneck unmodified; earlier versions floored them at $10^{-3}$ s^−1^ as a guard against division by zero, but no reported quantity actually divides by the rate, every value involved is representable in double precision, and the floor was silently raising long-haul fibre rates by up to twelve orders of magnitude (the model gives $1.5\times 10^{-12}$ s^−1^ at 3000 km, which the floor reported as $10^{-3}$)—it has since been removed. End-to-end rate is the bottleneck hop rate multiplied by $p_{\mathrm{swap}}^{\;n_{\mathrm{hops}}-1}$, aggregated across pairs as the mean of log(1+R) rather than the logarithm of the mean. Finally, a decoded topology with fewer than five edges is rejected outright and the oracle returns the fixed penalty vector $\left(\bar{F},{\bar{R}}_{\log},\bar{L},C_{\tau }\right)=\left(0.5,0,1.0,0\right)$, as it does when no pair is connected at all; both cases are dominated by every topology reached in practice, so neither affects the reported front.

All satellite parameters follow the Micius-calibrated regime cited above, and weather, elevation-angle dependence, orbital availability windows beyond the aggregate duty cycle δ, and adaptive optics are all left unmodelled. That last point is more than a caveat to note in passing: every link budget term in Equation (A8) ($T_{\mathrm{atm}}$, pointing loss, duty cycle) stays constant right up to $d_{\max}^{s}$ and then the link simply disappears, whereas a real link degrades continuously as elevation falls toward the horizon—so links near $d_{\max}^{s}$ are systematically more optimistic than links near the sub-satellite point. How much of the front this actually touches can be quantified directly: of the 1333 city pairs within visibility, 104 (7.8%) sit in the outer 10% of the horizon-limited range and 221 (16.6%) in the outer 20%, and among the satellite edges the pooled evolutionary front’s underlying topology pool actually uses, the corresponding shares are 4.9% and 12.4%. Imposing a minimum-elevation constraint (say 5–$20^{{}^{\circ}}$) would pull $d_{\max}^{s}$ in below the zero-elevation value used here and shrink this optimistic tail; we leave quantifying that effect on the front to future work. FSO links have the mirror-image problem: a hard 150 km range limit with no explicit curvature or elevation check, even though a symmetric, unobstructed path at that distance would actually require terminals roughly $d^{2}/\left(8R\right)\approx 0.44$ km above both endpoints, which the model simply does not represent. Because only 11 of the 4950 city pairs fall within FSO range in the first place, the exposure is small and enumerable; those 11 pairs are better read as a corridor with implicit elevated relays or towers than as a validated ground-level free-space link.

### Appendix A.5. Path-Search Implementation Validation

The exact-search claim of Section 4.2 is checked three independent ways, beyond the algebraic substitution identity given there. First, agreement between the oracle’s Dijkstra-like relaxation and an independent −logw shortest-path implementation over 18,364 reachable pairs of the paper’s own decoded topologies (maximum absolute fidelity difference $3.3\times 10^{-16}$, at the level of double-precision rounding, not a residual discrepancy). Second, agreement of both implementations with exhaustive enumeration of all simple paths on 200 random 7-node graphs (3925 pairs, maximum absolute difference 0 and $2.2\times 10^{-16}$ respectively), which is small enough to brute-force directly and so provides a ground truth independent of either search algorithm. Third, the decoder’s edge-inclusion behaviour (the effective per-pair probability of Section 4.1) is checked against a direct Monte-Carlo simulation of the decoding process rather than trusted from the combinatorial argument alone—e.g., at p=0.5, realised inclusion frequencies of 0.752 for mutual *k*-nearest-neighbour pairs and 0.501 for one-sided pairs, against the predicted 0.75 and 0.50 (decoder_edge_probability_v3.py; decoder_edge_probability_v3.json). All three checks are released with the reproducibility package.
